# Wearables in ADHD: Monitoring and Intervention—Where Are We Now?

**DOI:** 10.3390/diagnostics15182359

**Published:** 2025-09-17

**Authors:** Mara-Simina Olinic, Roland Stretea, Cristian Cherecheș

**Affiliations:** 1 Clinical Hospital for Infectious Diseases, 400348 Cluj-Napoca, Romania; mara.simi.olinic@elearn.umfcluj.ro; 2 Department of Neurosciences, Psychiatry and Pediatric Psychiatry, Faculty of Medicine, Iuliu Hațieganu University of Medicine and Pharmacy, 400012 Cluj-Napoca, Romania; 3 Scattered Technologies SRL., 400417 Cluj-Napoca, Romania; cristian@crescient.ro

**Keywords:** ADHD, wearables, personalized medicine, monitoring, intervention, artificial intelligence

## Abstract

**Introduction:** Wearable devices capable of continuously sampling movement, autonomic arousal and neuro-electrical activity are emerging as promising complements to traditional assessment and treatment of Attention-Deficit/Hyperactivity Disorder (ADHD). By moving data collection from the clinic to everyday settings, these technologies offer an unprecedented window onto the moment-to-moment fluctuations that characterise the condition. **Methods:** Drawing on a comprehensive literature search spanning 2013 to February 2025 across biomedical and engineering databases, we reviewed empirical studies that used commercial or research-grade wearables for ADHD-related diagnosis, monitoring or intervention. Titles and abstracts were screened against predefined inclusion criteria, with full-text appraisal and narrative synthesis of the eligible evidence. A narrative synthesis was conducted, with inclusion criteria targeting empirical studies of wearable devices applied to ADHD for monitoring, mixed monitoring-plus-intervention, or intervention-only applications. No quantitative pooling was undertaken due to heterogeneity of designs, endpoints, and analytic methods. **Results:** The reviewed body of work demonstrates that accelerometers, heart-rate and electrodermal sensors, and lightweight EEG headsets can enrich clinical assessment by capturing ecologically valid markers of hyperactivity, arousal and attentional lapses. Continuous monitoring studies suggest that wearable-derived metrics align with symptom trajectories and medication effects, while early intervention trials explore haptic prompts, attention-supporting apps and non-invasive neuromodulation delivered through head-worn devices. Across age groups, participants generally tolerate these tools well and value the objective feedback they provide. Nevertheless, the literature is limited by heterogeneous study designs, modest sample sizes and short follow-up periods, making direct comparison and clinical translation challenging. **Conclusions:** Current evidence paints an optimistic picture of the feasibility and acceptability of wearables in ADHD, yet larger, standardised and longer-term investigations are needed to confirm their clinical utility. Collaboration between clinicians, engineers and policymakers will be crucial to address data-privacy, equity and cost-effectiveness concerns and to integrate wearable technology into routine ADHD care.

## 1. Introduction

Attention-Deficit/Hyperactivity Disorder (ADHD) is among the most common pediatric psychiatric disorders, with prevalence estimates around 5–7% in children globally [1]. In the United States, recent figures suggest that over 11% of school-aged children have received an ADHD diagnosis [2], and a substantial proportion of those individuals continue to experience symptoms into adulthood [3]. In Europe, medication usage has notably increased, potentially driven by heightened awareness and improved diagnostic methods [4]. ADHD also imposes a large economic burden, with annual costs in the United States alone exceeding $120 billion, primarily attributable to unemployment-related expenses and productivity losses [5]. ADHD poses substantial mental health challenges, often co-occurring with conditions such as anxiety, depression, bipolar disorder, personality disorders, and substance use disorders. Individuals with ADHD also face an elevated risk of addiction, suicide, and low self-esteem. Beyond its psychological effects, ADHD significantly impacts daily life, contributing to academic difficulties, unstable employment, higher rates of delinquency and incarceration, and challenges in maintaining relationships. These struggles frequently lead to lower overall quality of life, affecting mental, social, and economic well-being [6]. Although stimulant and behavioral therapies remain foundational, their limitations—ranging from side effects to resource-intensive delivery—motivate the exploration of complementary digital and wearable interventions. Additionally, the positive impacts of behavioral therapies observed in controlled research settings do not always translate into daily life improvements without continued support [7].

ADHD assessment remains multifactorial and context-dependent. Presentations vary across the lifespan and settings; symptoms frequently co-occur with or are mimicked by sleep disturbance, anxiety/depression, learning difficulties and autism-spectrum traits [6]; current pathways depend on clinical interview and multi-informant rating scales that are vulnerable to recall and expectancy biases [8]. No single biomarker is recommended in routine care, and differential diagnosis often requires longitudinal corroboration across home, school and work environments. Treatment, while anchored in stimulant and behavioral approaches, is complex: a meaningful subset experiences partial or unstable response, dose-limiting side effects, adherence barriers, or limited transfer of clinic gains to daily functioning [6,8].

In this context, wearable sensors are best viewed as complements to clinical assessment. Their objective, continuous and time-stamped measures can reduce reliance on subjective, retrospective reports [9] and may accelerate key steps in care by: enabling earlier triage (improved screening), streamlined differential diagnosis using ecologically valid signals (e.g., activity, autonomic arousal, sleep/circadian regularity, and, in some cases, electroencephalography), symptom prediction and risk stratification [9,10,11], supporting individualized titration and longitudinal follow-up—often with remote, low-burden monitoring [12,13], and informing non-invasive intervention approaches (e.g., biofeedback, neurofeedback, and cueing via smartwatch apps) [14,15,16].

This review examines the landscape of wearable-based strategies, assessing both diagnostic and therapeutic applications. The objective of this work was to organize a broad and rapidly evolving literature on wearables for ADHD into a field-mapping framework rather than to compute a single summary effect size. Because devices, endpoints, and analytic pipelines vary widely, a narrative synthesis supported by a comprehensive search was considered methodologically appropriate. Representative performance metrics are reported to provide a general frame of reference, while acknowledging that direct pooling across heterogeneous outcomes would risk misleading inference. This positioning is intended to help readers locate evidence, gaps, and translation pathways across monitoring, mixed, and intervention-only trajectories.

## 2. Physiology and Behavioral Pathology of ADHD

Attention-Deficit/Hyperactivity Disorder (ADHD) is increasingly recognized as a neurodevelopmental condition resulting from a combination of genetic and environmental factors that influence both brain-wide networks and specific neurotransmitter systems [17] (8). ADHD pathophysiology stems from perturbations at both the molecular and systems levels: dopaminergic and noradrenergic dysregulation can disrupt frontostriatal circuits and default mode network connectivity, thereby influencing core domains of motivation, arousal regulation, and executive function [18,19,20]. Imaging studies have consistently linked ADHD symptoms to atypical connectivity patterns within and between these networks, such as diminished segregation between the DMN (default mode network) and task-positive networks, leading to attentional lapses and difficulties suppressing internal “mind-wandering” during tasks [18].

On the molecular level, ADHD has long been associated with dopaminergic and noradrenergic dysregulation, partly evidenced by the efficacy of stimulant medications that increase synaptic availability of these catecholamines, often ameliorating core symptoms [21,22]. However, recent genome-wide association studies indicate that risk variants for ADHD extend beyond dopamine-related genes, involving pathways crucial for early brain development, synaptic plasticity, and other neurotransmitter systems such as glutamate [23]. This broader perspective emphasises the high heterogeneity within the ADHD population and the necessity of adopting dimensional, rather than strictly categorical, approaches to capture the varied neurobiological processes driving symptom presentation and persistence [24,25].

Clinically, ADHD is characterized by persistent patterns of inattention, hyperactivity, and impulsivity, all of which disrupt everyday functioning and developmental progress. Inattention typically manifests as difficulty sustaining focus on routine tasks, with individuals prone to becoming easily distracted by either external stimuli or internal thoughts [26]. At times, these deficits coexist with hyperfocus episodes, wherein the individual becomes absorbed in particularly stimulating activities (e.g., gaming) to the detriment of other obligations. Further compounding these attention problems are impairments in task planning, organization, and decision-making—often resulting in missed deadlines, overlooked details, or scheduling conflicts [6]. Hyperactivity and impulsivity in ADHD manifest as restlessness—evidenced by fidgeting, tapping, or excessive talking—and a tendency to act or speak without considering consequences Whereas children may display overt physical restlessness, adults often experience a more internalized restlessness or persistent mental agitation [8]. Impulsivity can also emerge through interrupting conversations, impulsive spending, or taking social risks. Emotional dysregulation, including heightened irritability and frustration, frequently accompanies these patterns, further complicating social and occupational adaptation [6].

Because these dysregulations manifest in physiological markers—such as heart rate variability (autonomic arousal) and EEG changes (cortical excitation), wearables can effectively capture real-time indicators linked to the core neurobiology of ADHD. Researchers have increasingly turned to a variety of sensor-based technologies to develop more personalized and adaptive interventions. In this framework, sensor-based biomarkers can be systematically classified according to the primary physiological functions they evaluate.

### 2.1. Autonomic Function and Emotional Regulation

Heart activity is commonly tracked to assess arousal and stress, through various parameters, the most used being electrocardiography (ECG), heart rate (HR), heart rate variability (HRV), and photoplethysmography (PPG). Electrocardiogram (ECG) sensors (often in chest straps) directly measure the heart’s electrical signals, yielding precise heart rate and heart rate variability (HRV) data [9]. Photoplethysmography (PPG) sensors (common in smartwatches) optically measure blood pulse waves at the wrist [27], providing continuous heart rate and HRV. These metrics reflect autonomic nervous system activity—for example, ADHD is linked to autonomic nervous system (ANS) dysregulation, and studies measure HR/HRV to detect differences in arousal or the impact of stimulant medications [28]. In practice, both ECG and PPG have been used: chest straps offer accuracy but only capture heart signals (ECG), whereas modern wearables like Fitbit watches passively collect heart data via PPG along with other metrics [9].

Breathing-sensor–based biofeedback (often termed respiratory biofeedback) employs noninvasive strain gauges—typically elastic bands positioned around the chest or abdomen—to continuously monitor respiratory rate and pattern (e.g., depth, interval, abdominal versus thoracic breathing) [14]. In children with ADHD, this physiological data is conveyed in real time, frequently via visual, auditory, or haptic feedback, enabling them to learn diaphragmatic, slow-paced breathing patterns aimed at triggering a relaxation response. Rooted in operant conditioning principles, this technique encourages self-regulation of autonomic processes, which are often dysregulated in ADHD [15].

EDA (Electrodermal Activity) sensors measure changes in skin conductance to assess sweat gland activity. This is a proxy for sympathetic nervous system arousal—higher EDA indicates increased stress or emotional excitement [28]. In ADHD applications, EDA can capture moments of heightened arousal, frustration, or stress that accompany tasks and social interactions. For instance, a wearable EDA sensor can detect stress responses during focus tasks or emotional outbursts, providing an objective signal of internal states [29]. EDA biofeedback might also help ADHD individuals learn to self-regulate anxiety and predict attentional states in order to improve focus [30].

**Figure 1:**
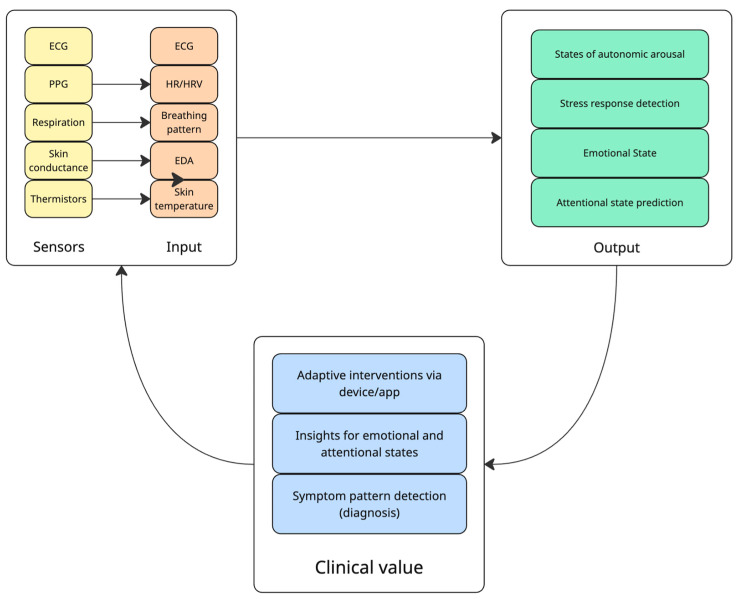
Sensors for physiological parameters.

Skin temperature sensors (thermistors or infrared thermometers) track peripheral temperature, which can shift with stress, activity, or circadian phase [28]. For example, a rising skin temperature combined with low activity at night might indicate the wearer is settling to sleep, whereas a sudden drop with sweating could signal anxiety [31]. Some devices also estimate respiration rate, either via chest expansion sensors or by analyzing PPG waveform variability [32]. A diagram illustrating the logical flow of extracting clinical value from sensors is present in Figure 1.

### 2.2. Motor and Physical Activity

**Figure 2:**
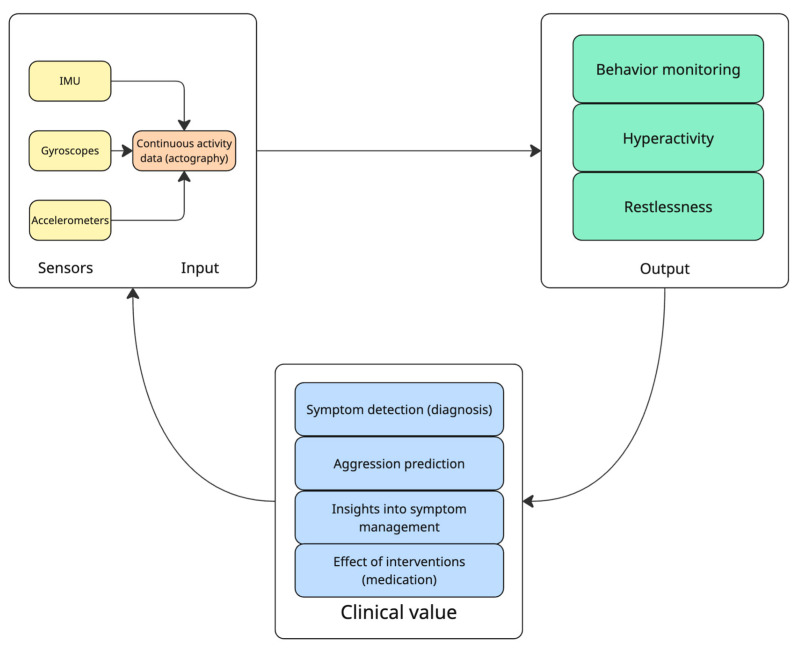
Sensors for movement and activity.

Motion sensors (Figure 2) are central to ADHD wearables, as they objectively capture hyperactivity, fidgeting, and sleep-restlessness. Accelerometers (typically 3-axis) measure linear movement and acceleration, enabling tracking of how much and how often a person moves. Gyroscopes measure angular velocity (rotation), adding detail on orientation and quick movements. All wearable studies targeting ADHD have relied on accelerometry to quantify motor activity [9]. These sensors record continuous activity data (often termed actigraphy when used for behavior monitoring). Motion sensors are small and low-power, easily embedded in wearables from wristbands to clip-on units. Many consumer devices (smartwatches, fitness trackers) contain a 6-axis inertial measurement unit (3-axis accelerometer + 3-axis gyroscope) [9].

Motion sensors in wearables enable objective monitoring of hyperactivity by tracking activity levels throughout the day, helping to identify periods of restlessness and assess the effects of interventions. These sensors detect variations in movement patterns, providing data that can be used to evaluate treatment effectiveness by measuring changes in activity levels and motor variability over time, offering valuable insights into ADHD symptom management [12].

**Figure 3:**
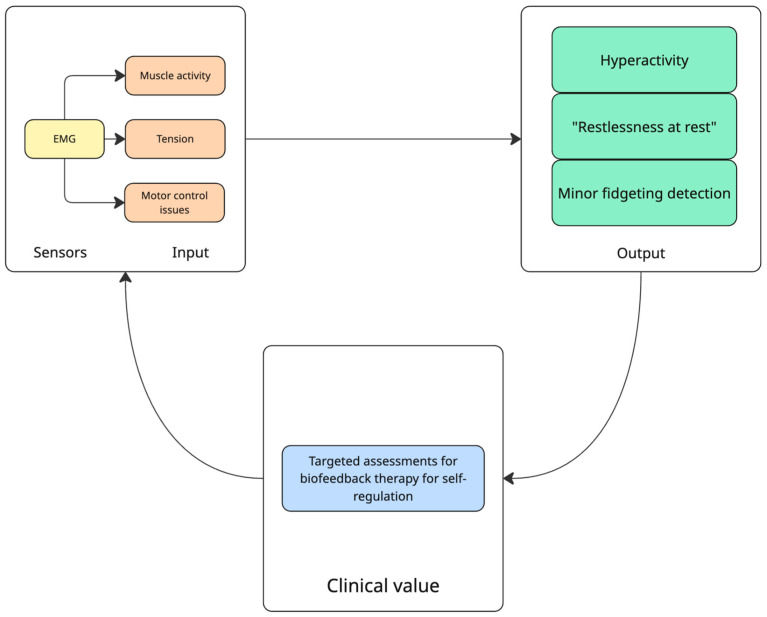
EMG sensors.

EMG (electromyography) sensors (Figure 3) measure muscle activity and are used to assess hyperactivity, muscle tension, and motor control issues in ADHD. Unlike accelerometers, which track larger movements, EMG is sensitive to subtle muscle activity, including slight tension that does not result in visible motion [33]. This allows it to detect the “restlessness at rest” often observed in ADHD—when an individual appears still but experiences internal tension or minor fidgeting. However, EMG requires adhesive electrodes placed on specific muscles, making it more complex to use than a standard actigraphy watch. As a result, its primary applications are in controlled settings, such as targeted assessments or biofeedback therapy, rather than continuous, long-term monitoring. Research indicates that individuals with ADHD frequently exhibit heightened baseline muscle tension and irregular motor patterns, which EMG can quantify objectively [34]. Additionally, EMG-based biofeedback has been explored as a potential intervention, showing promise in helping individuals manage restlessness and improve self-regulation [35].

### 2.3. Neurocognitive Function and Brain Activity

To monitor attention and brain-state in ADHD, some wearables incorporate electroencephalography (EEG) sensors. EEG sensors use electrodes placed on the scalp (or embedded usually in headbands to detect the brain’s electrical activity. They measure voltage differences generated by neural currents, providing a window into brainwave patterns [36]. Modern wearable EEG headsets use dry electrodes and wireless transmission, making EEG monitoring more practical outside the lab. These devices typically have fewer electrodes than clinical EEG (e.g., 2–8 channels versus 19+ in lab setups), but they still capture key frequency bands (alpha, beta, theta, etc.) [37]. Integrating EEG with other sensors can enhance accuracy—for instance, combining EEG attention metrics with physiological signals yielded high accuracy (around 89–91%) in distinguishing high vs. low mental workload in one study [27].

Electrooculography (EOG) sensors measure eye movements by detecting the electrical potential between the cornea and retina [38]. In ADHD, EOG sensors have been utilized to monitor attention levels, diagnose attention deficits, and differentiate between individuals with ADHD and healthy controls, while also being integrated into wearable devices for real-time tracking of attention [39,40].

### 2.4. Sleep and Circadian Rhythms

Sleep problems are frequently comorbid with ADHD—many children with ADHD have irregular sleep schedules, insomnia, or disturbed sleep architecture [6]. Wearable sensors help track sleep patterns and circadian rhythms in ADHD unobtrusively. The primary sensor used for wearable sleep monitoring is the accelerometer (actigraphy) [9]. In addition to motion, heart rate sensors (PPG) are now commonly used to enhance sleep tracking. Many smartwatches and rings combine accelerometer data with PPG-derived heart rate variability to detect sleep stages (light, deep, REM—rapid eye movement) and overall sleep stress [32]. Meta-analyses confirm that actigraphy can detect greater motor activity and sleep disturbances in ADHD compared to controls. ADHD youth exhibit longer sleep onset latency, more nighttime movement, and lower sleep efficiency on actigraphic recordings than non-ADHD peers [41].

## 3. Materials and Methods

An integrative narrative synthesis was undertaken to map and interpret heterogeneous evidence on wearable technologies in Attention-Deficit/Hyperactivity Disorder (ADHD). This design was selected owing to diversity in sensors, endpoints, study designs, and analytic pipelines that precludes quantitative pooling and favors contextual interpretation across modalities and settings.

### 3.1. Sources and Search Strategy

A broad literature search was conducted in PubMed, Scopus, Web of Science, IEEE Xplore, and the Cochrane Library. The search was last accessed on 17th of June 2025 and targeted publications from 2013 to the present, thus encompassing contemporary methods and devices. A representative Boolean strategy combined ADHD terms with wearable-technology and sensor modalities in titles/abstracts (e.g., (“ADHD” OR “Attention Deficit Hyperactivity Disorder”) AND wearable AND (EEG OR neurofeedback OR “sensor” OR “body movement” OR accelerometry OR HRV OR “heart rate” OR biometrics OR “motor activity” OR “haptic feedback” OR sleep OR “executive function” OR “behavior monitoring” OR attention)). Reference lists of pertinent articles were hand-searched to identify additional reports. Records were managed and deduplicated in a reference manager before relevance screening.

### 3.2. Eligibility Criteria and Scope

This review focused on peer-reviewed, empirical studies in humans examining wearable technologies for ADHD. Populations were defined by a clinical ADHD diagnosis according to DSM/ICD or by validated ADHD instruments. “Wearable device” was operationalized as a body-worn sensorized system capturing physiological and/or behavioral signals via on-body hardware (e.g., accelerometers/IMUs, photoplethysmography for HR/HRV, electrodermal activity, EEG); smartphone-only or desktop/web applications without physical sensor hardware were not considered wearable. Eligible devices comprised on-body sensors capable of continuous or near-continuous acquisition (accelerometry/IMU, PPG-derived HR/HRV, EDA, EEG), irrespective of market positioning (consumer vs. research-grade), to maximize ecological validity and representativeness of the evidence base.

Outcomes of interest encompassed standardized clinical measures (e.g., ADHD symptom ratings, executive-function tests) and objective sensor-derived indices (e.g., HRV metrics, accelerometry features, EEG parameters), reported across naturalistic (home, school, community) and controlled (clinic/laboratory) settings.

In terms of study types, emphasis was placed on randomized trials, quasi-experimental designs, open-label pilots, feasibility studies, and technical validations with human participants. The scope intentionally did not extend to theoretical overviews, simulation-only work, grey literature or preprints, or non-English publications.

After consolidating records across databases and applying the scope above, 31 reports were retained as central to the aims of this review, including one report of an ongoing clinical trial relevant to emerging practice. A consolidated summary of these studies is provided in Supplementary Tables S1–S3.

### 3.3. Evidence Charting and Synthesis

Data were charted for population characteristics, setting, device class and sensors, study design, targeted clinical domains (e.g., inattention, hyperactivity/impulsivity, executive function, sleep/circadian, arousal/emotion), outcome measures, and any reported quantitative indices (e.g., area under the curve, standardized mean differences/Cohen’s d, mean changes). Selection and data charting were performed by one reviewer and cross-checked by a second author. To preserve intent heterogeneity, findings were organized along three trajectories: (i) monitoring-only, (ii) monitoring-plus-intervention, and (iii) intervention-only. Quantitative indices are reported descriptively to illustrate observed ranges; no statistical pooling or model-based aggregation was attempted.

### 3.4. Appraisal of Methodological Features

Methodological features—sampling and allocation procedures, blinding where applicable, outcome ascertainment, follow-up completeness, feasibility/adherence—were appraised qualitatively to contextualize claims of efficacy or utility. No formal risk-of-bias scoring or meta-analytic weighting was performed, consistent with the narrative design. To aid interpretability within a narrative framework, each study was assigned a qualitative, study-level Methodological concern rating—Low, Moderate, or High—reflecting selection/confounding, outcome measurement validity, missing data/attrition, adherence/feasibility, and sample size/follow-up.

### 3.5. Methodological Limitations

This narrative approach is susceptible to selection and reporting biases; the restriction to English-language, peer-reviewed articles may have omitted relevant work. Database coverage, indexing differences, and variable reporting standards may have limited retrieval of some studies. Substantial heterogeneity in devices, analytic methods, and outcome definitions constrained comparability and precluded meta-analysis. No protocol was prospectively registered, in line with the narrative scope.

Generative-AI tools were employed exclusively for post-production tasks—namely spelling, grammar and syntax correction as well as graphic layout. No generative system was used to create or modify the scientific text, interpretations or statistical analyses presented in this manuscript.

## 4. Recent Advances in Wearable Technologies for ADHD

Modern wearable devices integrate multiple sensors, including electrodermal activity (EDA), heart rate variability (HRV), motion sensors, and electroencephalography (EEG), to capture a wide range of physiological and behavioral signals relevant to ADHD. These technologies enhance diagnostic accuracy and provide objective, real-time measures of the challenges individuals with ADHD face. Biosensor-based wearables monitor physiological indicators such as HRV, EDA, and skin temperature, offering insights into stress levels, emotional arousal, and autonomic regulation, which are valuable for both clinical assessment and diagnosis. Movement-tracking devices equipped with accelerometers, gyroscopes, and inertial measurement units (IMUs) objectively measure hyperactivity, capturing data on physical activity, sleep patterns, and even subtle fidgeting. Meanwhile, EEG-based wearables are emerging as a promising tool for monitoring brain activity, providing real-time data on attention fluctuations that can support both diagnostic evaluations and therapeutic interventions.

### 4.1. Monitoring Features of Wearables Devices in ADHD

A notable proportion of included studies employed consumer-grade wearables, reflecting their prevalence in community-based and pragmatic research settings. Fitbit and Apple Watch are among the most widely used devices for ADHD monitoring. Fitbit, in particular, has been extensively utilized in research due to the Adolescent Brain Cognitive Development (ABCD) research consortium which collected from nearly n = 12.000 adolescents a dataset incorporating genetic, demographic, and environmental data as well as a comprehensive range of measures assessing predictors and outcomes related to both mental and physical health, available at the National Institute of Mental Health data repository [42]. Kim et al. conducted a diagnostic study utilizing subsets of wearable data from the ABCD study to predict Attention-Deficit/Hyperactivity Disorder (*n* = 1090) and sleep problems in children (*n* = 3414) [43]. The study developed machine learning (ML) models based on circadian rhythm–derived features from Fitbit wearable wrist trackers. Participants included 79 children diagnosed with ADHD, 68 with sleep problems, and their respective control groups (1011 for ADHD and 3346 for sleep problems). The wearable data spanned 21 days and included metrics such as heart rate, sleep stages, and physical activity. Machine learning models (Random Forest, XGBoost, and LightGBM) were employed to classify ADHD and sleep problems, with performance evaluated using AUC (area under the receiver-operating-characteristic curve), sensitivity, specificity, PPV (positive predictive value), and NPV (negative predictive value). The LightGBM model demonstrated strong predictive performance for ADHD (AUC = 0.798) and sleep problems (AUC = 0.737), with heart rate variables being key predictors for ADHD, and sedentary activity and nap duration significant for sleep problems, indicating moderate-to-good discrimination from passively collected wearable features for ADHD prediction. Rahman et al. advanced this work by analyzing Fitbit-derived sedentary time, resting heart rate, and energy expenditure in a larger ABCD sample (*n* = 450). Correlation analyses showed significant associations between these Fitbit metrics and ADHD diagnosis. Multivariable logistic regression identified key predictors: higher resting heart rate and greater energy expenditure were positively associated with ADHD, while increased sedentary time predicted lower odds of diagnosis. Random Forest achieved cross-validation accuracy of 0.892, test accuracy of 0.888, precision of 0.88, recall of 0.90, F1-score of 0.89, and AUC of 0.95, consistent with excellent case–control separation and clinically meaningful discrimination [10].

Denyer et al. (2025) [13] extended this line of research by examining sleep patterns over a 10-week period in 20 individuals with ADHD and 20 controls using Fitbit Charge 3 devices. While mean sleep parameters—duration, onset, offset, and efficiency—did not differ between groups, those with ADHD exhibited significantly greater night-to-night variability across all sleep measures [13]. The study conveyed magnitude via non-significant t-tests for mean-level differences and highly significant F-tests for greater night-to-night variability in sleep duration, onset/offset, and efficiency among ADHD participants. No within-person associations were observed between sleep variability and fluctuations in anxiety or depressive symptoms. These findings suggest that sleep irregularity—rather than average sleep quality or quantity—may serve as a distinctive digital marker in ADHD.

Other smaller scale study (*n* = 30) have used newer models such as Fitbit Charge 4, demonstrating high predictive accuracy for ADHD classification. In this feasibility sample, classification performance was moderate-to-strong when using objective wearable signals alone (AUC = 0.844) and improved when combined with questionnaire data (AUC = 0.933), while medication-status discrimination was perfect in-sample (AUC = 1.000); standardized effects such as Cohen’s d or Pearson’s r were not reported [44].

Apple Watches have also been utilized for the objective assessment of ADHD symptoms. Two studies were conducted by Arakawa et al. and Lindheim et al. using the LemurDx app on Apple smartwatches to measure hyperactivity in children aged 5 to 12 years and 6 to 11 years, respectively. Arakawa et al. study presents within-sample discrimination of 85.2% accuracy (F1 = 81.6%) with parent-labeled context and 82.0% accuracy (precision = 76.9%, recall = 80.0%, F1 = 78.4%) using automatically inferred context under leave-one-participant-out validation, suggesting promising signal detection. Lindheim et al. pilot study’s performance indices (accuracy 0.89; sensitivity 0.93; specificity 0.86) suggest promising signal detection, but the absence of AUC and standardized effect sizes leaves the magnitude of effect and generalizability less precisely characterized [45,46].

Among the devices not intended for general consumer use, Muñoz-Organero et al. employed Runscribe inertial sensors from Scribe Labs, featuring four tri-axial accelerometers placed on the wrists and ankles. These sensors collected 24-h acceleration data, which was subsequently analyzed using a Recurrent Neural Network (RNN), effectively identifying distinct movement patterns, successfully distinguishing between children with ADHD and typically developing controls [47].

While the use of physiological signals as standalone indicators for Attention-Deficit/Hyperactivity Disorder has been relatively limited, recent studies have begun to explore their diagnostic potential. For instance, Andrikopoulos et al. (2024) [28] conducted a case-control study involving 76 participants (32 with ADHD and 44 healthy controls) where participants performed Stroop tests while wearing multi-sensor devices that recorded electrodermal activity (EDA), heart rate variability (HRV), and skin temperature (ST). Utilizing a Support Vector Machine (SVM) classifier, the study achieved a balanced accuracy of 81.6%, with sensitivity and specificity rates of 81.4% and 81.9%, demonstrating the potential of multimodal physiological signals as reliable markers for detecting adult ADHD [28].

An important aspect of ADHD behavior is aggression, a prevalent issue with potentially devastating consequences for the social integration of individuals. Park et al. (2023) [11] explored the potential of wearable-sensor-derived physical activity data combined with machine learning to objectively identify physically aggressive incidents. Their study involved 39 children aged 7 to 16 years, both with and without ADHD, who wore the ActiGraph GT3X+ waist-worn activity monitor. By leveraging machine learning, they achieved over 80% precision, accuracy, and recall in detecting aggression episodes [11].

Several wearable systems combine passive monitoring with prompting to support symptom management. For example, Revibe Connect is a wrist-worn device with embedded six-axis inertial sensors (accelerometer and gyroscope) paired with a smartphone application that summarizes activity-derived metrics. In an open-label, single-arm, 4-week pilot in unmedicated children with ADHD, parent ratings indicated a large within-group improvement in inattention (d = 1.07) and a moderate–large reduction in hyperactivity/impulsivity (d = 0.70), while teacher ratings suggested more modest gains in inattention (d = 0.54). Given the absence of a comparator and unblinded outcome assessment, these estimates should be regarded as preliminary signals that require confirmation in adequately powered, randomized, blinded, and controlled trials [48]. CASTT (Child Activity Sensing and Training Tool) takes a more advanced approach compared to previous projects that relied on a single wearable device by integrating multiple wearable sensors and interfaces to enhance monitoring capabilities. The authors distill design criteria for real-time assistive tech in classrooms across three components—sensing, recognizing, assisting—based on school observations/interviews with teachers and ADHD professionals. The system i [49] heart rate monitor, accelerometers placed on the arms and feet to track movement, and an Electroencephalogram (EEG) device connected via a smartphone [50].

A growing subset of ADHD-focused wearables uses electroencephalography (EEG) to capture real-time brainwave activity related to attention, hyperactivity, and impulsivity. These devices have been employed for both passive monitoring—detecting lapses in attention [40]—and active neurofeedback interventions, in which users learn to modulate specific brainwave frequencies to improve concentration [15,51]. EEG devices are primarily used for diagnostic purposes and continuous monitoring of brainwave activity to assess attention patterns and neurophysiological markers associated with the disorder [52,53,54,55]. Across these studies, wearable/EEG-based approaches show promising discrimination for ADHD: multimodal or ensemble models in preschoolers reach ~0.95–0.97 accuracy, while a web-app case study reported 100% accuracy in children and 90% in adults [49,55]. The task-evoked EEG study found significant alpha/theta coherence differences between ADHD and typical development but did not report classifier metrics [53]. Integrating wearable electroencephalography (EEG) with behavioral assessments has led to the development of a practical, multi-domain model that enhances the diagnosis of ADHD in young children, achieving a sensitivity of 92.3% and a specificity of 90.0% [49]. Prabhu et al. propose an innovative smart wearable headband prototype that integrates EEG and EOG sensors, allowing for real-time monitoring of brain activity and eye movements during tasks, enabling the identification of subtle changes in alertness, attention, and perception, that shows promising applications also in diagnosing ADHD, epilepsy and sleep disorders [40]. A summary list of studies and main findings regarding diagnostic and monitoring devices is presented below (Table 1).

### 4.2. Interventional Applications of Wearable Technologies

To address the need for non-pharmacological approaches, interventional applications utilizing wearable devices for Attention-Deficit/Hyperactivity Disorder (ADHD) management have become increasingly prevalent, offering several advantages such as portability, real-time feedback, and continuous monitoring outside traditional clinical environments. Key applications include self-regulation, improving focus and attention, managing hyperactivity and stressful states, or overall symptom reduction.

#### 4.2.1. Behavioral Interventions

Researchers have designed both ADHD-specific devices, as well as applications used with commercially available smartwatches. One of these is represented by StopWatch, an Apple Watch application for improving attention. The app utilizes actigraphy to track movement and provided visual and haptic feedback during focus sessions. Thirty-two participants aged 8–17 years were recruited, and ADHD symptoms were assessed using the ADHD Rating Scale (ADHD-RS). The results showed significant improvements in ADHD symptoms, with a weekly reduction of 1.2 units on the ADHD-RS, adding up to ~7 points decrease over 6 weeks, from an implied baseline of 32.5/54—sum of reported subscale means (95% CI: 0.56 to 1.88, *p* = 0.0004). Both inattentive and hyperactive/impulsive subscales demonstrated improvement. Children used the watch’s haptic and visual feedback to manage hyperactivity, especially in classroom contexts, though teachers’ feedback was not fully blinded [16]. FOQUS is another smartwatch application (tested on Samsung Gear 2) designed to assist adults with ADHD and attention-related challenges through behavioral management techniques. The app incorporates three core features: a flexible implementation of the Pomodoro technique to support focus and task completion, guided mindful meditation with visual and haptic cues for stress reduction, and positive message priming to enhance motivation and emotional well-being. Usability tests with 10 participants aged 21–30 over a seven-day period revealed that 80% of users reported reduced stress and anxiety after meditation sessions, with features such as inhale/exhale vibration cues being particularly effective during desk-based activities. Although some concerns were noted regarding heart rate measurement accuracy, participants valued the instant feedback provided by the app. Objective ADHD symptom improvement was not extensively measured [56].

Other devices and systems designed to help individuals with ADHD improve focus and attention are Revibe Connect and CASTT (Child Activity Sensing and Training Tool). Revibe Connect provides subtle, real-time cues to redirect attention and enhance self-regulation. The device collects both active and passive data, such as focus rates, fidgeting, and activity levels, which are analyzed using AI to offer personalized insights and interventions. Additionally, it includes features like text reminders to support executive functioning and a companion app for tracking progress and providing motivational rewards. An open-label pilot study evaluated the wearable device for improving attention, executive function, and academic performance in children with ADHD, showing significant parent- and teacher-reported improvements in attention, self-monitoring, and school functioning after four weeks of use. While preliminary, these results indicate promise for time-sensitive haptic reminders in real-world settings [57]. Further evidence for the intervention is anticipated from additional results of a completed randomized controlled trial evaluating three investigational wearable digital therapies [58].

As presented earlier, CASTT uses a group of wearable sensors and interfaces to provide real-time assistance in regaining attention for children. A preliminary study involving 20 children—both with and without ADHD—tested the system in a school environment. Findings suggest that real-time tracking of physical and physiological activity may be beneficial. However, challenges were noted, including discomfort caused by wearing multiple sensors and occasional missed notifications [50].

Further efforts to support behavioral interventions for children with ADHD have explored the use of wearable devices to foster self-awareness and behavioral regulation through real-time feedback. Garcia, De Bruyckere, Keyson, and Romero (2013) [59] developed two such devices: KITA (Kinesiofeedback Toy for ADHD), a tangible user interface designed for children aged 4–7, which utilizes an accelerometer to measure activity levels and provides vibration feedback to indicate whether movements are within acceptable limits, and WRISTWIT, a smartwatch-like wearable for children aged 8–12, designed to enhance time awareness and independent work by displaying time through LED indicators while monitoring body movements. Both devices were developed using a user-centered design approach that included children with ADHD and their caregivers, ensuring their suitability for school settings. Preliminary pilot studies conducted over two days demonstrated a reduction in activity levels, suggesting that these devices may offer valuable support for behavioral regulation in children with ADHD [59].

#### 4.2.2. Biofeedback Mechanisms

Doppel is a wrist-worn wearable device designed to provide heartbeat-like vibrations to the wrist, aiming to reduce anxiety and improve focus. The vibrations can be adjusted to either calm the user (slower vibrations) or enhance alertness (faster vibrations), leveraging principles of biofeedback. A double-blind, randomized controlled trial, over 8 weeks, aimed to evaluate Doppel’s efficacy in reducing anxiety and improving focus among young adults with ADHD. Forty-nine participants aged 18–25 years were assigned to either an active Doppel group or a comparator group. The active Doppel group received vibrations linked to their heart rate, while the comparator group received fixed vibration patterns. While both groups demonstrated significant reductions in anxiety and improvements in focus over time, no significant differences were observed between the active Doppel and comparator conditions, indicating that the benefits may not be specific to heart rate-linked vibrations [60].

Emotional regulation difficulties are a known risk factor for social challenges and comorbidities, such as anxiety, depression, and substance abuse in young individuals with ADHD [6]. Researchers have addressed these challenges by designing a wrist-worn device that integrates sensors for Galvanic Skin Response (GSR), pulse rate (PR), and skin temperature (ST). These physiological markers are utilized to detect variations in mood or stress. The device incorporates an Artificial Intelligence model capable of predicting emotional instability. When such instability is anticipated, the device provides a warning on the wrist unit, enabling individuals to recognize and better manage their emotional state [29]. A summary list of studies and main findings regarding intervention and monitoring devices is presented below (Table 2).

#### 4.2.3. Neuromodulation

Among the unique wearable technologies reviewed, trigeminal nerve stimulation (TNS), as explored in a study by McGough et al., represents an innovative non-invasive neuromodulation method designed specifically for managing ADHD symptoms. Unlike consumer-focused smartwatches or fitness trackers, TNS targets the trigeminal nerve via a small device worn during sleep, delivering low-level electrical currents through electrodes placed on the forehead. This approach directly engages brain regions involved in attention regulation, such as the reticular activating system and anterior cingulate cortex. Conducted as a double-blind, sham-controlled trial, 62 children aged 8–12 years were randomized to either active TNS or sham treatment for four weeks, followed by an additional week without treatment to assess response persistence. The primary outcome measure, the ADHD Rating Scale (ADHD-RS), showed significant improvement with active TNS compared to the sham group (Cohen’s d = 0.50), demonstrating a medium treatment effect size. Clinical Global Impression-Improvement (CGI-I) scores also favored active TNS, with a number needed to treat of 3. Resting-state quantitative EEG revealed increased spectral power in right frontal and frontal midline frequency bands with active treatment, correlating with reductions in hyperactive and impulsive symptoms. No clinically meaningful adverse events were reported, confirming the safety and tolerability of TNS. The study demonstrates TNS’s efficacy in reducing ADHD symptoms with a medium treatment effect size comparable to non-stimulant medications, alongside improvements in brain spectral power linked to hyperactivity and impulsivity. Its minimal risk and high tolerability further distinguish TNS as a novel addition to ADHD treatment modalities [61].

Wearable Brain-Computer Interface (BCI) integrated with Augmented Reality (AR) smart glasses have been studied for ADHD rehabilitation. The device utilizes single-channel EEG instrumentation to detect Steady-State Visual Evoked Potentials (SSVEP), enabling children to remotely control a robot through visual stimuli and eye blinks. This system was tested on individuals with ADHD and demonstrated high user acceptance, sustained engagement, and improved attentional performance [62,63,64].

A randomized, controlled, parallel-group design study was conducted to evaluate the efficacy of a novel feed-forward modeling (FFM) system as a nonpharmacological intervention for children with ADHD. Participants, aged 8–12 years, engaged in 24 sessions of FFM-based cognitive training over 6–8 weeks, utilizing an EEG headband (Zeo Sleep Manager™) to monitor and adapt the intervention in real time. The training incorporated a video game-like environment where EEG feedback informed gameplay, requiring participants to regulate their attention and impulse control. Significant improvements in ADHD symptomatology and academic performance were observed in the FFM group compared to the control group, which received standard nonpharmacological care. Furthermore, these gains were sustained three months post-intervention, highlighting the FFM system’s potential as a durable, first-line treatment for ADHD, particularly in enhancing attention and academic outcomes [65].

Emerging technologies, such as the Novostim 2 device, are advancing the field of ADHD treatment through innovative approaches. Currently undergoing a randomized, sham-controlled clinical trial, Novostim 2 employs transcranial random noise stimulation (tRNS), a non-invasive technique that applies weak electrical signals to the brain to enhance its natural activity. Designed for pediatric patients aged 7–12 years, the device delivers 20-min stimulation sessions over a two-week period, accompanied by a digital engagement game to maintain attention. The trial aims to evaluate the device’s safety and efficacy as a non-pharmaceutical intervention for ADHD, with the ultimate goal of obtaining FDA clearance [66]. Santamaría-Vázquez et al. implemented a combined intervention that integrated electroencephalographic (EEG) neurofeedback with respiratory biofeedback—where slow diaphragmatic breathing was guided via audio-visual cues—and incorporated active median nerve stimulation (MNS) delivered through a wrist-worn device, compared against a sham-MNS control. Sixty children with ADHD underwent this protocol, and analyses revealed significantly increased frontal theta power (*p* = 0.0125)—a neurophysiological change associated with symptom reductions in anxiety, hyperactivity, impulsivity, and inattention—that persisted at a one-month follow-up (*p* = 0.0325), with EEG changes correlating with improvements in clinical rating scales [15].

#### 4.2.4. Peripheral Visual Stimulation

Yael Richter and colleagues conducted a two-month, open-label study to evaluate the safety and efficacy of Neuro-glasses, a novel intervention using peripheral visual stimulation, for managing ADHD symptoms in adults. The study aimed to assess whether this sensory-based approach could reduce inattentive symptoms and improve executive functioning [67].

The researchers enrolled 108 adults aged 18–40 with a documented ADHD diagnosis. Participants were provided with personalized Neuro-glasses featuring semi-transparent stimuli embedded in the lenses and were instructed to wear them for at least two hours daily. ADHD symptoms were assessed at baseline and post-intervention using self-reported measures (Adult ADHD Self-Report Scale—ASRS and Behavior Rating Inventory of Executive Function–Adult—BRIEF-A), a computerized test of attention and impulsivity (CPT-3), and clinician ratings (CGI-I). The results demonstrated significant improvements in inattentive symptoms (ASRS inattentive index, *p* = 0.037) and metacognitive functions related to self-management and performance monitoring (BRIEF-A, *p* = 0.029). The CPT-3 revealed enhanced target detectability (d’, *p* = 0.027) and reduced commission errors (*p* = 0.004), indicating better response inhibition. Clinician ratings showed that 62% of participants experienced clinically meaningful improvements (CGI-I). The intervention was well-tolerated, with no serious adverse events reported [67].

### 4.3. Real-Time Data Collection and Integration with Digital Platforms

Real-time data collection enables the immediate capture of contextually rich insights into ADHD-related symptoms, facilitating more responsive and individualized interventions. Modern wearables leverage continuous sensing, meaning that heart rate variability, electrodermal activity, or movement data can be captured throughout the day rather than at isolated time points, enabling the timely identification of fluctuations in attention, hyperactivity, and stress [22,53]. This real-time approach has sparked interest in ecological momentary assessment (EMA), a method involving the collection of real-time data on individuals’ behaviors, thoughts, and emotions within their natural environments, thereby minimizing recall bias and enhancing ecological validity [68]. This real-time feedback allows for prompt adjustments to therapeutic or educational strategies, addressing acute symptom variations as they occur. Furthermore, continuously collected data often provides a more accurate representation of an individual’s typical behavioral and physiological patterns, leading to deeper insights into their everyday experiences. By enhancing self-awareness, such feedback enables individuals to identify and manage potential triggers for inattention, restlessness, and stress. In the context of remote or virtual treatment models, continuous monitoring supports informed clinical decision-making without necessitating frequent in-person consultations [7].

A crucial component of any modern wearable-based system is how the collected data are processed, shared, and ultimately acted upon. Integration with digital platforms—such as mobile apps or cloud dashboards—can substantially enhance the utility of wearable devices for both clinical and everyday contexts [69]. Most ADHD-focused wearables transmit captured data (e.g., heart rate variability, daily step counts, or device usage logs) to a companion mobile application [16,29,45,50,57,70]. This connectivity facilitates the comprehensive visualization of ADHD-related data, providing clinicians and caregivers with actionable insights for more effective decision-making. For instance, the Apple Watch application developed for ADHD delivers visual and haptic feedback on movement levels, enabling users to track and analyze symptom progression over time [16]. Additionally, cloud-based systems support the continuous collection and analysis of health data, offering a holistic perspective on the patient’s condition and enabling timely interventions [71]. By improving the precision of ADHD symptom monitoring and fostering better communication among patients, caregivers, and healthcare providers, these technologies contribute to a more coordinated and effective approach to managing the disorder [9]. A summary list of studies and main findings regarding intervention-only devices is presented below (Table 3).

## 5. Efficacy and Challenges

### 5.1. Clinical Outcomes

Recent advancements in ADHD-focused wearable technologies and other digital interventions have yielded promising—though often preliminary—clinical outcomes in managing core attention and hyperactivity/impulsivity symptoms. Evidence now encompasses a breadth of study designs, ranging from pilot work and feasibility demonstrations to controlled investigations—highlighting both the growing sophistication of wearable sensors and apps as well as a persistent need for larger, more rigorous trials.

A recurrent motif across these studies is the high diagnostic accuracy attained by sensor-based monitoring systems when differentiating individuals with ADHD from neurotypical cohorts. For instance, multiple research groups have used smartwatch data (e.g., Fitbit, Apple Watch) to train machine learning models, often achieving balanced accuracies or area-under-the-curve (AUC) values above 0.80 [43,44,45,46,72]. Accelerometry, heart rate variability (HRV), and electrodermal activity (EDA) represent key physiological signals that are readily captured, and integrated datasets appear to boost diagnostic yield. One case-control study employing multi-sensor wearables during neuropsychological testing reported balanced accuracy above 80% in distinguishing adults with ADHD from controls [28]. Children and adolescents using aggression-monitoring algorithms—integrated via waist-worn ActiGraph monitors—demonstrated that machine learning methods could detect aggression episodes in real time with over 80% accuracy [11]. Going further, emotional instability can be anticipated using wearable devices, enabling individuals to recognize and better manage their emotional state [29]. These findings raise the prospect of more personalized, context-aware interventions across a range of symptom domains.

On an interventional front, novel devices such as Neuro-glasses have shown beneficial effects on inattentive symptoms and executive functioning in open-label studies, with reported improvements in continuous performance task accuracy and clinically meaningful change rates exceeding 60% [67]. Clinical trials of nonpharmacological approaches have similarly grown in scope. A double-blind, sham-controlled study of trigeminal nerve stimulation (TNS) documented medium effect sizes (Cohen’s d ≈ 0.50) for ADHD symptom improvement after four weeks of nightly stimulation, marking TNS as a well-tolerated complement or potential alternative to medication in some cases [61]. Meanwhile, interventions incorporating wearable EEG headsets or real-time feedback mechanisms, though still limited by sample size constraints, have reported promising short-term gains in executive function or parent-rated hyperactivity [50,64,65].

An increasing number of pilot projects also target outcomes beyond core ADHD symptomatology. Studies of watch-based prompts (e.g., FOQUS, Revibe Connect) suggest short-term growth in organization, self-monitoring, or classroom on-task behavior. Specific devices (e.g., Revibe Connect) [57] or smartwatch apps like FOQUS [56] and StopWatch [16] have been designed to deliver in-the-moment reminders, mindfulness exercises, or haptic prompts for maintaining focus. Preliminary data from a wearable Apple Watch application (StopWatch) showed weekly ADHD-RS declines of −1.2 points/week in a six-week open-label trial, highlighting the feasibility of leveraging haptic and visual smartwatch feedback to reduce hyperactivity in situ [16]. The study investigating Revibe Connect demonstrated statistically significant improvements in attention, self-monitoring, and academic functioning following a four-week intervention, based on reports from both parents and teachers. However, as an open-label study, the findings are subject to potential reporting bias, which may influence the overall efficacy [57].

Nevertheless, cross-study comparisons remain challenging due to variations in sensor modalities, outcome measures, and data analysis methodologies. To facilitate clinical integration and enhance the reproducibility of findings across trials, future research should prioritize the establishment of standardized protocols for device calibration, measurement endpoints, and data security. Preliminary usability findings suggest that most participants perceived these wearable-based tools as engaging and beneficial for short-term stress reduction and task completion [16,56,60,63]. However, large-scale evaluations and the incorporation of more objective outcome measures remain limited.

### 5.2. Barriers to Adoption

Although recent work suggests encouraging outcomes from wearable and digital tools for ADHD, a number of challenges continue to limit widespread use in real-world settings. On the technical side, constraints on battery life can hinder continuous data collection, while potential inaccuracies of biosignal measurements (for example, electrodermal activity or heart rate variability) pose concerns about robust, real-time data capture [11,16]. Achieving adequate signal fidelity without compromising ergonomics or aesthetics remains a notable hurdle, especially for younger users who may reject devices deemed bulky or invasive [50,73]. These factors can lead to diminished acceptance over time if the technology fails to align seamlessly with daily routines.

Ethical and privacy issues also represent important barriers. Wearable systems continuously track personal physiological and behavioral information, prompting apprehensions about data security, user consent, and the potential for unauthorized use [9]. Parents may be reluctant to adopt such tools unless there are clear guidelines on encryption, secure data protocols, and child-friendly consent processes [74]. Systems that offer classroom-based monitoring risk stigmatizing children if they are perceived as visibly singled out [50].

Another significant challenge concerns cost, which can be prohibitive for families with restricted financial means or inconsistent healthcare coverage. While consumer devices (for instance, Fitbit or Apple Watch) may be more widely available, ongoing expenses—including subscription fees and proprietary sensor parts—can compound disparities [75]. Furthermore, the absence of insurance coverage or reimbursement for novel ADHD interventions reduces their accessibility, particularly for historically underserved communities [76]. Addressing these limitations calls for collaborative efforts among manufacturers, healthcare professionals, and policymakers to enhance affordability, sustainability, and cultural acceptance. Lastly, the integration of wearable devices into clinical workflows remains limited. Many clinicians are not well-versed in the interpretation of wearable-generated data, or question the reliability of analytics that have not undergone extensive validation [47,56]. Consequently, pediatricians, psychologists, and educators may hesitate to incorporate these tools without established outcome data or standardized operating procedures. Partnerships involving device developers, telehealth services, and frontline practitioners could help incorporate validated sensor-based diagnostics and interventions into clinical practice in a more systematic way.

### 5.3. Clinical Translation, Regulatory Pathway and Market Access Strategy

A significant barrier to adoption is the pathway that every device needs to follow in order to be approved and recognized as a medical device. We discuss several relevant jurisdictions with their own regulatory strategies.

#### 5.3.1. United States

For wrist-worn ADHD wearables comprising sensors plus Software as a Medical Device (SaMD), classification and claims should be scoped first (monitoring vs. therapeutic adjunct); this determines risk class and evidence needs. Early alignment with FDA is typically sought via the Q-Submission (Q-Sub) program to clarify classification, predicate suitability (for 510(k)), or the need for a De Novo request when no predicate exists, as well as clinical endpoints, human-factors expectations in minors, and cybersecurity documentation [77]. Premarket files are expected to follow the FDA guidance on Content of Premarket Submissions for Device Software Functions (software documentation tiers, hazard analysis, verification/validation), with clinical evidence framed to the IMDRF/FDA SaMD Clinical Evaluation approach (valid clinical association, analytical validation, and clinical validation). If any clinician-facing logic is supplied, boundaries with Clinical Decision Support (CDS) policy should be mapped to determine whether elements qualify as non-device CDS. Cybersecurity design controls (secure architecture, SBOM, vulnerability handling, update strategy) are addressed per FDA’s current premarket cybersecurity guidance. Adaptive/AI models are generally aligned to FDA/IMDRF Good Machine Learning Practice concepts and emerging change-control principles.

#### 5.3.2. European Union

Under Regulation (EU) 2017/745 (MDR), the software component of an ADHD wearable is typically qualified and classified under Rule 11; the sensor may be an accessory in a system. A manufacturer QMS (ISO 13485 [78] or equivalent) is implemented; Annex I General Safety and Performance Requirements are addressed in the technical documentation; and a Clinical Evaluation demonstrates performance and benefit–risk in the intended population (including concomitant ADHD medication use where relevant). Conformity assessment via a Notified Body is required for Class IIa or higher. Post-market activities include UDI assignment, EUDAMED registration, PMS/PMCF planning, and cybersecurity risk treatment [79]. Harmonized standards commonly applied include ISO 14971 [80], IEC 62304 [81], and IEC 62366-1 [82] for risk, software life-cycle, and usability; the MDCG guidance on software qualification/classification is used to justify Rule 11 application and borderline determinations [83].

#### 5.3.3. Cost-Cutting Strategies

Costs are typically reduced by (i) scoping initial claims to monitoring/trend reporting and deferring higher-risk therapeutic features to later iterations; (ii) aligning design controls to ISO 14971/IEC 62304/IEC 62366-1 from project inception to avoid remediation; (iii) using early regulator dialogue (Q-Subs; NB scientific advice) to lock endpoints and study design; (iv) planning AI/ML change control once (GMLP-consistent) for multi-region filings; and (v) designing trials and data operations deliberately to satisfy payer coding/coverage criteria (e.g., documentation that supports service billing in routine care). In the US, Remote Physiologic Monitoring (RPM) requires automated device data capture and, for device supply/collection codes, at least 16 days of readings in a 30-day period (not applicable to management-time codes). Program design (enrollment, clinician time capture, and device logistics) is typically aligned to these requirements [84]. Remote Therapeutic Monitoring covers therapeutic/functional data flows and management time; it can include patient-reported or device-captured data, with therapist and physician billing routes pre-defined. For innovative products, Medicare coverage strategy may include the Transitional Coverage for Emerging Technologies (TCET) pathway, which offers time-limited, evidence-development–aligned coverage for a small number of FDA-designated Breakthrough devices that fit existing Medicare benefit categories, with an aim to finalize a national coverage decision shortly after authorization [85]. In the EU, reimbursement is national and heterogeneous; several structured routes can be targeted first to fund evidence and scale. For example, in Germany, CE-marked Class I/IIa SaMDs may obtain statutory insurance coverage through the DiGA Fast-Track program, following a 3-month BfArM assessment of quality (e.g., data protection/interoperability/usability) and proof of positive healthcare effect; provisional listing is available while confirmatory evidence is completed. Analyses of DiGA listings show how evidence and outcomes are typically framed [86]. Pan-European syntheses highlight the emerging convergence and persistent fragmentation in assessment and reimbursement for digital medical devices, which informs sequencing of evidence across countries [87].

### 5.4. Future Directions

Innovations in sensor miniaturization, machine learning algorithms, and cloud-based analytics promise to enhance the accuracy and applicability of wearables for ADHD management. Future device iterations may incorporate multimodal data—integrating EEG, ECG, EDA, and accelerometry—to bolster both diagnostic and interventional precision [27,28,54]. The incorporation of adaptive machine learning models, capable of learning an individual’s unique physiological and behavioral signatures, holds potential for more personalized interventions that respond to real-time fluctuations in attention, stress, and hyperactivity [38,67].

Importantly, next-generation wearables may move beyond mere monitoring to deliver tailored cognitive training, guided behavioral prompts, or neurofeedback protocols, seamlessly integrated into day-to-day routines. The integration of these advanced features with telemedicine platforms or ecological momentary assessments (EMA) could catalyze a paradigm shift—enabling truly individualized, data-driven ADHD care that transcends the walls of clinical settings [9,65,66]. Robust longitudinal research and randomized controlled trials will be required to further validate these emerging capabilities across diverse demographic and clinical subgroups.

## 6. Risks and Ethical Considerations

The potential benefits of wearable technology for ADHD must be weighed against critical risks. One concern involves overreliance on continuous feedback, which may undermine users’ self-regulatory capacity once the device is removed [74]. Intensive monitoring risks pathologizing normal behavioral fluctuations, prompting excessive vigilance from individuals with ADHD and their caregivers regarding relatively minor shifts in physiological metrics.

### 6.1. Algorithmic Bias

Algorithmic bias raises further ethical challenges. Machine learning models often rely on samples drawn from primarily Western, higher-income settings, which may yield misclassifications or reduced accuracy when applied to populations with different sociocultural backgrounds [9]. Such disparities can affect diagnostic precision and intervention outcomes, disproportionately impacting members of racial and ethnic minority groups, lower-income households, or those with comorbid conditions.

To minimize the risk that models trained predominantly on Western, higher-income cohorts perform poorly in under-represented populations, future studies should: (i) broaden recruitment beyond these settings and routinely report performance stratified by age, sex, ethnicity, socioeconomic status, and comorbidity [9]. (ii) conduct external validation across independent sites and countries and explicitly evaluate domain shift (e.g., device brand/firmware, sampling rates, wear-time habits, school schedules) [88,89] (iii) address representation and measurement bias at the data level through prespecified inclusion criteria and sensor data quality control (non-wear and artifact detection, time alignment, harmonized sampling), complemented by reweighting or oversampling under-represented subgroups and devices [69]; (iv) implement model-level safeguards—probability calibration, subgroup-aware decision thresholds [88], periodic audits with model cards—and, where data cannot be centralized, privacy-preserving training (e.g., federated learning, differential privacy) and (v) publish transparent error analyses (e.g., confusion matrices and failure modes by subgroup) to make clear where and why models underperform [88,89].

### 6.2. Pediatric Consent and Assent

Because minors cannot provide full legal consent, studies and deployments should pair parent/guardian consent with age-appropriate assent, using plain-language materials that explain what signals are collected (e.g., HRV, EDA, movement), who can access them, how long they are kept, and how to withdraw. Protocols should include data-protection impact assessment, encryption, and policies for incidental findings and school-based use to avoid stigmatization or coercion, with a designated contact point for families and periodic re-consent as adolescents’ capacities evolve. Importantly, HIPAA/GDPR compliance is necessary but may be insufficient for children; child-specific safeguards and governance are recommended [73,90,91].

For pediatric wearable data, a layered governance model that combines binding legal duties with operational standards is recommended. Under the GDPR, controllers should anchor processing in the core principles—lawfulness, fairness, transparency; purpose limitation; data minimization; accuracy; storage limitation; integrity/confidentiality; accountability—and treat children’s data as warranting specific protection; a Data Protection Impact Assessment should be conducted for high-risk, technology-mediated processing; and, where consent is the lawful basis, parent/guardian permission should be paired with age-appropriate child assent for information-society services [92]. In the U.S., services directed to children under 13—or those that knowingly collect from them—must comply with COPPA, including clear parental notices, verifiable parental consent before collection/use/disclosure, reasonable data-security safeguards, and retention/deletion limits tied to necessity [93]. To operationalize these duties, organizations can implement a Privacy Information Management System to embed privacy roles, risk assessment, processor oversight, and lifecycle controls; and use the NIST Privacy Framework to profile risks, set policies for data sharing, and align governance with engineering practice [94,95]. For child-centered design, the UK ICO’s Age-Appropriate Design Code (Children’s Code) offers widely adopted good practice: default-high privacy, data minimization, geolocation/profiling off by default, and plain-language transparency suited to the child’s age [96].

### 6.3. Data Privacy and Governance

Wearable systems frequently collect sensitive biometric data, including heart rate, emotional state, and sleep patterns, all of which hold considerable interest for both clinical and commercial entities. Strict compliance with encryption standards, transparent consent mechanisms, and clear communication about data usage practices is essential to uphold ethical standards and maintain user trust [69,90,97]. Compliance with HIPAA or GDPR alone may not suffice in pediatric populations where minors cannot legally consent, and guardians’ comprehension of technology usage and data sharing might vary. Clear disclaimers about how real-time location or biometric data may be used or monetized remain critical.

## 7. Limitations

This narrative synthesis has inherent limitations. Heterogeneity in samples, devices, firmware, endpoints, and analytic choices precluded quantitative pooling and limits generalizability. Several included studies leveraged consumer wearables. This expands reach, adherence, and scalability but introduces measurement heterogeneity. Follow-up durations were often short; wear-time adherence and missing-data strategies were variably reported; and external validation across devices and demographics was uncommon. Publication bias cannot be excluded. These constraints were anticipated and are the rationale for organizing results as a framework with representative metrics rather than attempting formal aggregation. Framing the literature as trajectories also clarifies translation steps: monitoring studies suggest candidate digital biomarkers for stratification and symptom tracking; mixed approaches motivate just-in-time interventions and adherence logic; intervention-only studies indicate where RCT-grade evidence is most needed (e.g., neuromodulation). This organization enables targeted methodological recommendations—common data elements, standardized wear-time and missing-data handling, and transparent model reporting—that are prerequisites for future meta-analytic synthesis. Subsequent work would benefit from: (i) consensus core outcome sets spanning clinical (e.g., ADHD-RS, executive-function tasks) and digital endpoints (e.g., activity volatility, HRV indices, sleep regularity); (ii) standardized reporting of sensor pipelines, wear-time thresholds, and missing-data handling; (iii) pre-registered, adequately powered multi-site studies with external validation across devices and age groups; and (iv) clear translation pathways (e.g., usability, human-factors, and regulatory readiness for mixed and intervention trajectories). Such steps would enable robust cross-study comparability and, ultimately, formal meta-analytic synthesis.

## 8. Conclusions

There is a growing body of research suggesting that wearable-based approaches, either as standalone tools or in conjunction with digital platforms, have the capacity to facilitate assessment and intervention for ADHD. Across multiple pilot investigations, wearable sensors have demonstrated modest yet meaningful improvements in measures of inattention or hyperactivity, as well as some gains in executive function or classroom engagement. However, the current evidence base is dominated by early-phase research, and only a handful of devices are supported by high-quality RCT data or regulatory clearance.

Several persistent barriers temper the promise of such technologies. Among the most pronounced are cost considerations, potential issues of data security, and the absence of clear implementation pathways in educational and clinical environments. Ethical concerns also arise, including the possibility of amplifying stigma when children wear conspicuous devices and the risk of machine learning models exhibiting bias against populations not well represented in study samples.

To strengthen the evidence base, large-scale, multisite trials using unified protocols and longer-term measurements of real-world outcomes are needed. Such investigations can clarify the characteristics of individuals most likely to derive benefit from wearable-assisted interventions, while also revealing best practices for integrating data from these devices into more traditional care models. Attention to barriers—particularly cost, privacy, and developer-clinician coordination—is critical for widespread uptake and equity of access.

Despite the obstacles and limited generalizability of current findings, wearables hold the potential to augment established ADHD treatments, offering feasible and context-sensitive means of improving self-regulation. Future research that thoroughly documents these technologies’ safety and efficacy over extended periods, while prioritizing inclusive design and safeguarding user privacy, will be central to confirming their long-term value in ADHD management.

## Figures and Tables

**Table 1:** Diagnostic/monitoring devices.

Clinical Target	Study	Objective	Sample	Study Design	Device/Sensors Involved	Results
Hyperactivity	Kim WP et al., 2023 [43]	ML prediction of ADHD/sleep problems from wearable data.	ABCD cohort subset; ADHD/controls: 79/1011; Sleep/controls: 68/3346.	Case-control ML classification (RF/XGB/LGBM); internal validation.	Fitbit (Google LLC) PPG HR/HRV, 3-axis accelerometer (activity/sleep)	ADHD: AUC 0.80; sens 0.76; spec 0.72; NPV 0.98. Sleep: AUC 0.74; sens 0.74; spec 0.63; NPV 0.99; heart rate strongest predictor
	Rahman MM et al. 2025 [10]	Fitbit-derived measures to predict adolescent ADHD via ML	ABCD cohort (release 5.0); *N* = 450 adolescents	Cross-sectional secondary analysis; logistic regression + ML classification with internal cross-validation (CV) and held-out test	Fitbit (Google LLC) PPG HR (resting HR), 3-axis accelerometer (activity/sedentary; energy expenditure derived)	RF (CV): AUC 0.95; acc 0.89; precision 0.88; recall 0.90; F1 0.89; held-out test acc 0.88; Fitbit metrics showed significant associations with ADHD in regression
	Jiang Z et al., 2024 [44]	Feasibility ML classification of ADHD and medication status from wearable actigraphy/HR in adolescents	ADHD/controls: 17/13; ages 16–17 (*N* = 30)	Longitudinal pilot; case–control ML (XGBoost) with internal validation	Fitbit (Google LLC) PPG HR; 3-axis accelerometer; actigraphy-derived sleep	ADHD (objective only): AUC 0.844; objective + subjective: AUC 0.933; medication-status classification: AUC 1.00. Key predictors: HR (resting/mean) & very active minutes (medication status); irritability/sex/QoL (ADHD)
	Lindhiem O et al., 2022 [45]	Objective measurement of hyperactivity in children using a smartwatch + ML (LemurDx)	*N* = 30 (ADHD-H/I or combined/controls: 15/15), ages 6–11; 2 days wear	Pilot observational case–control; supervised ML classification; usability assessed	Apple Watch (LemurDx app) 3-axis accelerometer (primary signal); contextual: heart rate, GPS, Bluetooth; App with parent input	Diagnostic accuracy 0.89; sensitivity 0.93; specificity 0.86 (with motion features + parent activity labels)
	Arakawa R et al., 2023 [46]	Objective hyperactivity measurement from smartwatch sensing (LemurDx)	Children 5–12 y; ADHD/controls: 25/36; *N* = 61; wear 2–7 days	Observational case–control ML classification; context filtering vs. none; leave-one-participant-out cross-validation (CV) for evaluation; 5-fold CV for hyperparameter tuning	Apple Watch (LemurDx app) 3-axis accelerometer (primary); HR (PPG), GPS, Bluetooth recorded for context (not used in final ML)	With parent-provided context filtering: AUC 0.85; acc 85.2%; F1 0.816. Without context: AUC 0.70; acc 67.2%; F1 0.630. Automated context (no parent input): acc 82.0%; F1 0.784. Threshold 0.505 → TPR 0.80, FPR 0.11. Slight correlation of risk score with VADPRS
	Muñoz-Organero M et al., 2019 [47]	Comparison (RNN-based) of movement patterns in ADHD vs. typically developing children from wrist/ankle accelerometry; medicated vs. non-medicated contrasts	*N* = 36; ADHD/controls: 18 (9 medicated, 9 non-medicated)/18; ages 6–16	Observational case–control; 24-h wear; RNN trained on 9 controls, evaluated on remaining 9 controls	Runscribe inertial sensors (Scribe Labs, CA, USA) 3i-axial accelerometers (wrists, ankles)	Non-medicated ADHD > “non-similar” fragments vs. controls: d ≈ 0.80 (estimated). Medicated vs. controls: d ≈ 0.50 (estimated)
Aggression/ agitation	Park C et al., 2023 [11]	ML detection of aggression episodes from waist-worn actigraphy in children with/without ADHD	*N* = 39; ages 7–16; repeated 1-week wear (3 times/12 months)	Observational monitoring; parent episode logs as labels; Random Forest model; internal validation	ActiGraph GT3X+ (ActiGraph Corp.)—triaxial accelerometer (waist)	AUC 0.893; accuracy 0.820; recall 0.850; precision 0.802; F1 0.824. Vector-magnitude acceleration higher during aggression vs. non-aggression (means 1580.7 ± 1831.1 vs. 873.3 ± 1137.2; approx. Cohen’s d ≈ 0.46, epoch-level; *p* = 0.027)
Attention/alertness/arousal	Chen IC et al., 2024 [49]	Multimodal ADHD detection in preschoolers using wearable EEG + behavioral measures	Preschoolers; ADHD/controls: 43/35 (*N* = 78)	Case–control ML/DL classification (Decision Tree/Random Forest/bi-LSTM); 5-fold internal validation; ensemble model	Wearable wireless EEG (Mindo BR8; 8-ch)	Ensemble accuracy 0.974 Sensitivity 92.3%, specificity 90.0% Effect sizes: K-CPT-2 HRT SD (ADHD 52.05 ± 8.45 vs. TD 47.94 ± 6.49) Cohen’s d ≈ 0.54; HRT ISI change (52.44 ± 8.89 vs. 47.69 ± 6.82) Cohen’s d ≈ 0.59
	Huang IW et al., 2024 [52]	Assess EEG complexity (parietal fuzzy entropy) to aid ADHD diagnosis	Children 4–7 y; ADHD/controls: 30/30	8-ch dry-EEG headband	8-channel wireless wearable EEG	Best feature set (right occipital beta PSD + parietal FuEn) achieved accuracy = 0.90;
	Lin JW et al., 2024 [53]	Characterize EEG functional-connectivity patterns—focusing on temporal alpha dissimilarity/coherence for potential diagnosis marker	N-72; Ages 8–16 y; ADHD/controls: 53/19	Case–control, task-evoked EEG study (visual CPT and auditory CATA tasks)	EEG sensors 16-ch EEG cap	Temporal-lobe FC in alpha during CATA was higher in TD vs. ADHD (*p* < 0.05).
	Santarrosa-López I et al., 2025 [55]	Develop and validate DETEC-ADHD, a web-based application that integrates machine learning with personal, clinical, psychological and EEG data to detect ADHD and its subtypes in real time.	N = 19 (Children n = 10; Adults n = 9; mixed ADHD and non-ADHD)	Proof-of-concept case study; Logistic Regression model	Webapp + Muse S headband (InteraXon)—EEG (dry electrodes)	Logistic Regression: accuracy = 90%; AUC = 0.92; case-study detection rates—children: 100%; adults: 90%.
Autonomic arousal	Andrikopoulos D et al., 2024 [28]	ML detection of adult ADHD from multimodal wearable signals during Stroop tasks	Adults; ADHD/controls: 32/44 (*N* = 76)	Case–control ML classification (LR/KNN/RF/SVM); internal cross-validation; i-KNN filtering, data collected during Stroop tests	Feel Monitoring Device + app (Feel Therapeutics)—EDA, PPG HR/HRV, skin temperature (9-axis IMU present; not modeled).	SVM (multimodal): accuracy 0.816; sensitivity 0.814; specificity 0.819. Unimodal models lower/less balanced
Sleep/ circadian rhythm	Denyer H et al. 2025 [13]	Remote 10-week monitoring of sleep in ADHD vs. controls; test group differences in mean vs. night-to-night variability and links with anxiety/depression.	*N* = 40 (ADHD/controls: 20/20), ages 16–39; 2428 nights total (median nights: ADHD 62; controls 68).	Observational non-interventional cohort; linear mixed models for mean sleep features	Fitbit Charge 3 (Fitbit/Google LLC)—3-axis accelerometer (sleep duration, onset, offset, efficiency)	ADHD showed greater night-to-night variability: SD duration 1:33 vs. 1:10; SD onset 2:02 vs. 1:43; SD offset 1:50 vs. 1:37; SD efficiency 4.23 vs. 3.67 (all *p* < 0.001); within-person anxiety/depression associations were non-significant
Monitoring medication	Ouyang CS et al., 2020 [12]	Objective evaluation of methylphenidate effects via smartwatch accelerometry	*N* = 10 children with ADHD (9M/1F); mean age ≈ 7.4 ± 1.3 y	Pre–post within-subject (baseline vs. 1-month methylphenidate 10 mg/day, weekdays); paired t-tests (Bonferroni α = 0.0167); correlation with SNAP-IV (teacher)	Garmin Vivosmart 3-axis accelerometer, HRV	Variance decreased after treatment: Y-axis 4.42 ± 2.17 → 2.32 ± 0.65 (*p* = 0.0119); Z-axis 4.09 ± 1.57 → 2.41 ± 0.81 (*p* = 0.0140). SNAP hyperactivity reduction correlated with Y-axis variance reduction (r = 0.605); other subscales weak/non-significant

ABCD—Adolescent Brain Cognitive Development cohort; acc—Accuracy; ADHD—Attention-Deficit/Hyperactivity Disorder; ADHD-H/I—ADHD Hyperactive/Impulsive subtype; AUC—Area Under the ROC Curve; bi-LSTM—Bidirectional Long Short-Term Memory (neural network); CATA—Continuous Auditory Test of Attention; CPT—Continuous Performance Test; CV—Cross-Validation; d—Cohen’s d (standardized mean difference); DL—Deep Learning; EDA—Electrodermal Activity; EEG—Electroencephalography; F1—F1-score (harmonic mean of precision & recall); FC—(EEG) Functional Connectivity; FPR—False Positive Rate; FuEn—Fuzzy Entropy; GPS—Global Positioning System; HR—Heart Rate; HRV—Heart-Rate Variability; HRT—(CPT) Hit Reaction Time; IMU—Inertial Measurement Unit; ISI—Inter-Stimulus Interval; i-KNN—instance-based/iterative k-Nearest Neighbors (filtering step); K-CPT-2—Conners’ Kiddie Continuous Performance Test, 2nd Edition; KNN—k-Nearest Neighbors; LGBM—Light Gradient Boosting Machine; LR—Logistic Regression; ML—Machine Learning; NPV—Negative Predictive Value; PPG—Photoplethysmography; PSD—Power Spectral Density; QoL—Quality of Life; RF—Random Forest; RNN—Recurrent Neural Network; SD—Standard Deviation; SVM—Support Vector Machine; TD—Typically Developing (controls); TPR—True Positive Rate; VADPRS—Vanderbilt ADHD Diagnostic Parent Rating Scale; XGB—Extreme Gradient Boosting (XGBoost).

**Table 2:** Monitoring and intervention devices.

Clinical Target	Study	Objective	Population	Study Design	Device/Sensors Involved	Key Findings
Multidomain clinical targets: attention, hyperactivity, impulsivity, executive function, behavior	Ayearst LE et al., 2023 [48]	Wearable digital intervention to improve on-task behavior—specifically attention, hyperactivity/impulsivity, executive function, and academic performance	ADHD, 8–12 y; *N* = 38 (parent raters *N* = 38; teacher raters *N* = 26); 4-week school wear; unmedicated	Single-arm, open-label pre–post pilot (4-week wearable use) in unmedicated children with ADHD; baseline → post parent/teacher ratings; no randomization, blinding, or control	Revibe Connect (Revibe Technologies)—haptic prompts; tap-back self-reports; step logging, 3-axis accelerometer, gyroscope	Parent ADHD-RS-5 inattention d = 1.07; hyperactivity/impulsivity d = 0.70. Teacher ADHD-RS-5 inattention d = 0.54. WFIRS-P school learning r ≈ 0.58 (large). APRS academic productivity d = 0.59 (moderate)
	Garcia JJ et al., 2013 [59]	Design-driven personal informatics (KITA/WRISTWIT) to support self-awareness and on-task behavior in ADHD	Children (KITA: 4–7 yrs *N* = 2, WRISTWIT: 8–12 yrs *N* = 5) Context informants *N* = 15	Empirical Research Through Design; iterative prototyping; in-situ school testing; exploratory sensing—no control group	KITA: waist-worn toy + “nest” (3-axis accelerometer; vibration motor; 31 LEDs; IR link; microcontroller/speaker in nest). WRISTWIT: bracelet (3-axis accelerometer; 12-LED time display)	KITA pilot: ~16% reduction in in-class activity vs. baseline; high engagement reported. WRISTWIT concept: accelerometry distinguished on-/off-task states; supports time awareness.
	Sonne T et al., 2015 [50]	Design and preliminary evaluation of CASTT—a real-time assistive wearable to help children with ADHD regain attention in school	Children 2nd-5tth grade (*n* = 20, ADHD/controls: 11/9)	Non-randomized, uncontrolled observational feasibility pilot study	CASTT (Child Activity Sensing and Training Tool) custom wearable + smartphone system: Heart rate monitor, accelerometers (limbs), EEG	CASTT was wearable in class and captured physical activity continuously in real time; preliminary evidence indicated practical feasibility in authentic school contexts.
	Santamaría-Vázquez E. et al. 2025 [15]	Test whether combined respiratory biofeedback, neurofeedback and median nerve stimulation improve ADHD symptoms	*N* = 60; ADHD randomized active group(AG)/sham group(SG): 31/29; ages 8–18;	Exploratory randomized, double-blind, sham-controlled, two-arm parallel trial; 10 sessions over 2 weeks; pre/post/1-mo follow-up; resting-state EEG	Qey-DTx NMS (median nerve stimulation) stimulator (wrist electrodes); ProComp Infiniti with respiration belt (breathing sensor); EEG Neuroamp II	Within-group improvements in AG post-treatment and at 1-mo follow-up (Cohen’s d: post—hyperactivity index −0.45, anxiety −0.34, impulsivity-hyperactivity −0.40; follow-up—learning −0.62, hyperactivity index −0.50, impulsivity-hyperactivity −0.53)
Attention, hyperactivity	Leikauf JE et al., 2021 [16]	Feasibility study of an Apple Watch app, tracking movement and delivering visual/haptic feedback to manage hyperactivity/attention in youth with ADHD	ADHD; *N* = 32; ages 8–17; 6-week follow-up	Open-label single-arm pilot; weekly ADHD-RS via parent report; linear mixed models for symptom trajectories; exit interviews (feasibility/acceptability)	Apple Watch Series 0 (Apple Inc., Cupertino, CA, USA) 3-axis accelerometer (actigraphy for movement); haptic motor (biofeedback)	ADHD-RS total β −1.2 units/week (95% CI −1.88 to −0.56; F = 13.4; *p* = 0.0004); Inattentive β −0.8/week (*p* = 7 × 10^−5^); Hyperactive/Impulsive β −0.4/week (*p* = 0.02); no adverse events; older age associated with greater improvement
Anxiety, arousal, emotional dysregulation	Dibia V, 2016 [56]	Smartwatch app (FOQUS) to support focus and reduce anxiety in adults with ADHD/attention difficulties	Survey n = 27 (ages 16–40) + 7-day usability study *n* = 10 (ages 21–30)	User-centred design; cognitive walkthrough + 7-day prototype usability test (no control)	Samsung Gear 2 (Samsung Electronics). PPG heart rate (pre/post meditation feedback); vibrotactile cues; positive-message priming	80% reported reduced stress/anxiety after meditation; observed HR decreases pre→post
	Whitehead JC et al., 2022 [51]	Remote EEG-neurofeedback efficacy for ADHD-related symptoms, cognition, and EEG markers	*N* = 593 (560 included), age > 13 Questionnaire pre–post *n* = 301; CPT pre–post *n* = 99 with known ADHD status (plus *n* = 104 unknown status); resting EEG baseline *n* = 271; pre–post EEG *n* = 41	Retrospective single-group pretest–posttest; home/clinic use	Muse EEG headband (InteraXon) via Myndlift app—4 dry electrodes	(Cohen’s d): questionnaires—Large pre–post improvements: ADHD-RS-IV abnormal d = 2.41, GAD-7 abnormal d = 1.24, PHQ-9 abnormal d = 1.13, ASRS abnormal d = 1.05, GHQ-12 abnormal d = 0.99; CPT—response-time variability d = 1.02–1.24, average RT d = 0.56 (healthy), commission d = 0.55–0.62, omission d = 0.34–0.48; EEG—baseline DAR higher in abnormal ASRS (d = 0.37); pre-post DAR reduced in abnormal group (d = 0.70)

ADHD-RS-5—ADHD Rating Scale, 5th Edition; AG—Active Group; APRS—Academic Performance Rating Scale; ASRS—Adult ADHD Self-Report Scale; β—Beta (regression slope/standardized coefficient); CASTT—Child Activity Sensing and Training Tool; CI—Confidence Interval; CPT—Continuous Performance Test; DAR—(EEG) Delta/Alpha power ratio; d—Cohen’s d; EEG—Electroencephalography; GAD-7—Generalized Anxiety Disorder 7-item scale; GHQ-12—General Health Questionnaire-12; HR—Heart Rate; IR—Infrared; LED—Light-Emitting Diode; NMS—Median Nerve Stimulation; PHQ-9—Patient Health Questionnaire-9; PPG—Photoplethysmography; r—Pearson correlation coefficient; RT—Reaction Time; WFIRS-P—Weiss Functional Impairment Rating Scale—Parent report.

**Table 3:** Intervention-only devices.

Clinical Target	Study	Objective	Sample	Study Design	Device/Sensors Involved	Key Findings
Overall ADHD symptom severity	McGough JJ et al., 2019 [61]	Non-invasive neuromodulation during sleep for symptom improvement in ADHD	Children 8–12 y; randomized: active/sham = 32/30 (N = 62)	Double-blind RCT; 4 weeks nightly eTNS + 1-week blinded discontinuation; weekly ADHD-RS & CGI; mechanistic qEEG	Monarch eTNS System (NeuroSigma): external stimulator with adhesive forehead patch electrodes	ADHD-RS: significant group × time (F(1, 228) = 8.12, *p* = 0.005); Cohen’s d = 0.50 at week 4. Clinical Global Impression-Improvement responders at week 4: 52% AG vs. 14% SG (NNT = 3). qEEG: increased frontal spectral power with active eTNS; partial r (EEG change ↔ ADHD-RS change) = −0.34 to −0.41
	McDermott AF et al., 2016 [65]	EEG feed-forward modeling (Atentiv/CogoLand) attention-training for pediatric ADHD; Neurofeedback training via EEG)	ADHD, 8–12 y; randomized: 46 (immediate FFM = 21; wait-list control = 19; total randomized = 46; 32M/14F)	Randomized parallel-group trial (8-week FFM vs. non-pharmacological community care), waitlist crossover; outcomes at post and 3-month follow-up	EEG headband with three frontal electrodes (Zeo Sleep Manager™) + PC game (CogoLand^®®^)	Clinician ADHD-RS: −36% vs. control; partial η^2^ (Group × Time) = 0.434. Parent ADHD-RS: −31%; partial η^2^ = 0.141. CGI: partial η^2^ (Group × Time) = 0.282. PERMP problems attempted: +26% (η^2^ > 0.150). Effects largely maintained at 3 months; Quotient^®^ (Pearson Education, Inc., Westford, MA, USA) ADHD no improvement
Attention, executive function	Richter Y et al., 2023 [67]	Evaluate efficacy of peripheral visual stimulation “Neuro-glasses” for adult ADHD	ADHD, 18–40 y; enrolled *N* = 108; per-protocol *N* = 97; wear ≥2 h/day	Open-label single-arm clinical trial; pre–post assessments (ASRS, BRIEF-A, CPT-3); CGI-I at endpoint	Neuro-glasses (Sparkles™, VIZO Specs Ltd., Tel Aviv, Israel)—standard lenses with semi-transparent peripheral stimuli; personalization with eye-tracking	ASRS-Inattention improved (*p* = 0.037), Cohen’s d = 0.22; BRIEF-A Metacognition improved (*p* = 0.029), d = 0.23; CPT-3 detectability d′ improved (*p* = 0.027), d = 0.23; CPT-3 commission errors reduced (*p* = 0.004), d = 0.30; 62% CGI-I responders
	Clinical trial NCT06189703 [66]	Examine the safety and effectiveness of tRNS on unmedicated pediatric patients	Children (7–12 yrs)	Randomized, sham-controlled, double-blind clinical trial	Novostim 2—Transcranial random noise stimulation device	Subjects will undergo either tRNS or sham treatment for 10 days during a two-week period in a home-simulated environment. Each treatment session is 20 min, during which their attention will be maintained using a software game.
Anxiety, focus	Bartlett G et al., 2024 [60]	Evaluate whether a wrist-worn haptic device (Doppel) reduces anxiety and improves focus in adults with ADHD over 8 weeks	Adults 18–25 y with self-reported ADHD; *N* = 49 at baseline; 4-week *n* = 37; 8-week *n* = 32 (active 14/comparator 18)	Double-blind randomized controlled trial; active HR-matched vibrations vs. fixed-pattern comparator; intention-to-treat	Doppel wristband + smartphone app; haptic actuator delivering heartbeat-like vibrations	No superiority of active vs. comparator at 4 or 8 wk (all *p* ≥ 0.31; partial η^2^ ≤ 0.03). Time effects across groups: anxiety ↓ (η^2^ = 0.10) and focus ↑ (η^2^ = 0.22)
Multidomain clinical targets: attention, hyperactivity, impulsivity, executive function, cognitive skills	Arpaia P et al., 2020 [62]	Wearable single-channel SSVEP BCI with AR glasses for robot-based rehabilitation in ADHD; evaluate accuracy/latency and feasibility	Algorithm tuning: *N* = 20 healthy adults; Robot-control test: *N* = 10 healthy adults; Clinical preliminary: *N* = 4 children with ADHD (6–8 y)	Instrumentation study + observational case study; training-less single-channel SSVEP with eye-blink detection; lab evaluation and rehab-center pilot	Epson Moverio BT-200 AR glasses (eye-blink detection); Olimex EEG-SMT (single-channel EEG); Sanbot Elf robot.	Accuracy–latency trade-off (e.g., 92.6% at ~3.71 s vs. 70.8% at ~0.64 s); clinical target setting selected ~1.5 s response time; case study average accuracy >83% with ITR up to 39 bits/min; preliminary ADHD tests reported positive acceptability/attentional engagement.
	Arpaia P et al., 2021 [63]	Wearable AR-based single-channel EEG (SSVEP) BCI to control a social robot for ADHD therapy; preliminary adherence evaluation	Children 5–10 y; *N* = 18 (ADHD); plus adult benchmark *N* = 10	Pilot case study (task-based robot control); descriptive outcomes on acceptance/adherence; no inferential testing.	Epson Moverio BT-200 AR glasses (eye-blink detection); Olimex EEG-SMT (single-channel EEG); Sanbot Elf robot.	Adherence: 18/18 accepted wearing; completion: all 8–10 y finished tasks; some 5–7 y had ergonomics/attention issues; prior adult test accuracy ≈83.5% for command detection
	Arpaia P et al., 2022 [64]	Evaluate a wearable EEG-based brain computer interface for rehabilitation/training ADHD therapy, assessing adherence and preliminary cognitive/attentional gains	Adherence/acceptability: *N* = 18 ADHD children; Therapy cohort: *N* = 7 ADHD children	Single-arm pilot (within-subject pre–post); task-based sessions (planning, path-following, inhibition) while controlling a social robot	Epson Moverio BT-200 AR glasses (eye-blink detection); Olimex EEG-SMT (single-channel EEG); Sanbot Elf robot.	High acceptability/adherence (18 screened). All 7 treated children showed improvement across BIA subtests after 1 month (e.g., higher semantic/phonological fluency, better visual-sequential and Span-4; fewer reading errors)

ADHD-RS—ADHD Rating Scale; AR—Augmented Reality; ASRS—Adult ADHD Self-Report Scale; BCI—Brain–Computer Interface; BRIEF-A—Behavior Rating Inventory of Executive Function—Adult version; CGI—Clinical Global Impression; CGI-I—Clinical Global Impression—Improvement; CPT-3—Conners’ Continuous Performance Test, 3rd Edition; d—Cohen’s d; d′—d-prime (signal-detection sensitivity index); EEG—Electroencephalography; eTNS—External Trigeminal Nerve Stimulation; FFM—Feed-Forward Modeling; HR—Heart Rate; ITR—Information Transfer Rate (BCI metric); NNT—Number Needed to Treat; PERMP—Permanent Product Measure of Performance; qEEG—Quantitative EEG; RCT—Randomized Controlled Trial; r—Pearson correlation coefficient; SSVEP—Steady-State Visual Evoked Potential; η^2^ (partial)—Partial eta-squared (effect-size measure).

## Data Availability

Not applicable.

## Supplementary Materials

The following supporting information can be downloaded at: https://www.mdpi.com/article/10.3390/diagnostics15182359/s1; Table S1: Studies about monitoring-only devices; Table S2: Studies about mixed-function devices (monitoring + intervention); Table S3: Studies about intervention-only devices.
