# Supplementary material for: Wearables in ADHD: Monitoring and Intervention—Where Are We Now?
Source: Diagnostics (Basel). 2025 Sep 17;15(18):2359. doi: 10.3390/diagnostics15182359 (PMC12468562; doi:10.3390/diagnostics15182359)
Supplement: Supplementary file 1 [file diagnostics-15-02359-s001.zip › diagnostics-3782878-supplementary.pdf]

Table S1. Studies about monitoring-only devices

Study	Objective	Symptom/Target	Sample	Study design	Device/Sensors involved	Results	Methodological concern
Kim WP et al., 2023 [47]	ML prediction of ADHD/sleep problems from wearable data.	Diagnosis - hyperactivity + sleep	ABCD cohort; ADHD/controls: 79/1011 ; Sleep/controls: 68/3346.	Case-control ML classification (RF/XGB/LGBM) ; internal validation.	Fitbit (Google LLC) PPG HR/HRV, 3-axis accelerometer (activity/sleep)	ADHD: AUC 0.80; sens 0.76; spec 0.72; NPV 0.98. Sleep: AUC 0.74; sens 0.74; spec 0.63; NPV 0.99 heart rate strongest predictor	Moderate - small ADHD case *N*; class-imbalance; no external validation
Rahman MM et al. 2025 [11]	Fitbit-derived measures to predict adolescent ADHD via ML	Diagnosis - hyperactivity	ABCD cohort (release 5.0); *N*=450 adolescents	Cross-sectional secondary analysis; logistic regression + ML classification with internal cross-validation (CV) and held-out test	Fitbit (Google LLC) PPG HR (resting HR), 3-axis accelerometer (activity/sedentary; energy expenditure derived)	RF (CV): AUC 0.95; acc 0.89; precision 0.88; recall 0.90; F1 0.89; held-out test acc 0.88; Fitbit metrics showed significant associations with ADHD in regression	Moderate - observational ABCD; potential confounding; unclear subject-wise split; class imbalance; no external validation
Denyer H et al. 2025 [48]	Remote 10-week monitoring of sleep in ADHD vs controls; test group differences in mean vs night-to-night variability and links with	Monitoring / Diagnosis - sleep	*N*=40 (ADHD/controls: 20/20), ages 16–39; 2,428 nights total (median nights: ADHD 62; controls 68).	Observational non-interventional cohort; linear mixed models for mean sleep features	Fitbit Charge 3 (Fitbit/Google LLC) — 3-axis accelerometer (sleep duration, onset, offset, efficiency)	ADHD showed greater night-to-night variability: SD duration 1:33 vs 1:10; SD onset 2:02 vs 1:43; SD offset 1:50 vs 1:37; SD efficiency 4.23 vs 3.67 (all p<.001); within-person anxiety/depression	Moderate - small *N*; consumer wearable; observational; adherence variable

	anxiety/depression.					associations were non-significant	
Jiang Z et al., 2024 [49]	Feasibility ML classification of ADHD and medication status from wearable actigraphy/HR in adolescents	Diagnosis - hyperactivity +/- subjective data	ADHD/controls: 17/13; ages 16–17 (*N*=30)	Longitudinal pilot; case–control ML (XGBoost) with internal validation	Fitbit (Google LLC) PPG HR; 3-axis accelerometer; actigraphy-derived sleep	ADHD (objective only): AUC 0.844; objective+subjective: AUC 0.933; medication-status classification: AUC 1.00. Key predictors: HR (resting/mean) & very active minutes (medication status); irritability/sex/QoL (ADHD)	Moderate - small *N* (30); 16–17 y only; external validation not reported; FU not specified
Lindhiem O et al., 2022 [50]	Objective measurement of hyperactivity in children using a smartwatch + ML (LemurDx)	Diagnosis - hyperactivity + parent labels	*N*=30 (ADHD-H/I or combined/controls: 15/15), ages 6–11; 2 days wear	Pilot observational case–control; supervised ML classification; usability assessed	Apple Watch (LemurDx app) 3-axis accelerometer (primary signal); contextual: heart rate, GPS, Bluetooth; App with parent input	Diagnostic accuracy 0.89; sensitivity 0.93; specificity 0.86 (with motion features + parent activity labels)	Moderate - small *N*; 2-day FU; internal CV; spectrum-restricted sampling
Arakawa R et al., 2023 [51]	Objective hyperactivity measurement from smartwatch	Diagnosis - hyperactivity +/- parent labels	Children 5–12 y; ADHD/controls: 25/36; *N*=61; wear 2–7 days	Observational case–control ML classification; context filtering vs. none; leave-one-participant-	Apple Watch (LemurDx app) 3-axis accelerometer (primary);	With parent-provided context filtering: AUC 0.85; acc 85.2%; F1 0.816. Without context: AUC 0.70; acc 67.2%; F1 0.630. Automated	Moderate - case–control; modest *N*; 2–7 days; noisy/estimated context

	sensing (LemurDx)			out cross-validation (CV) for evaluation; 5-fold CV for hyperparameter tuning	HR (PPG), GPS, Bluetooth recorded for context (not used in final ML)	context (no parent input): acc 82.0%; F1 0.784. Threshold 0.505 → TPR 0.80, FPR 0.11. Slight correlation of risk score with VADPRS	
Muñoz-Organero M et al., 2019 [52]	Comparison (RNN-based) of movement patterns in ADHD vs typically developing children from wrist/ankle accelerometry; medicated vs non-medicated contrasts	Diagnosis - hyperactivity	*N*=36; ADHD/controls: 18 (9 medicated, 9 non-medicated)/18; ages 6–16	Observational case–control; 24-h wear; RNN trained on 9 controls, evaluated on remaining 9 controls	Runscribe inertial sensors (Scribe Labs, CA, USA) 3i-axial accelerometers (wrists, ankles)	Non-medicated ADHD > "non-similar" fragments vs controls: d=0.80.<br>Medicated vs controls: d=0.50	Moderate - small *N*; single 24 h; convenience sample; mixed medication; partial significance
Andrikopoulos D et al., 2024 [31]	ML detection of adult ADHD from multimodal wearable signals during Stroop tasks	Diagnosis - physiological data (autonomic nervous system activation)	Adults; ADHD/controls: 32/44 (*N*=76)	Case–control ML classification (LR/KNN/RF/SVM); internal cross-validation; i-KNN filtering, data collected during Stroop tests	Feel Monitoring Device + app (Feel Therapeutics) — EDA, PPG HR/HRV, skin temperature (9-axis IMU present; not modeled).	SVM (multimodal): accuracy 0.816; sensitivity 0.814; specificity 0.819. Unimodal models lower/less balanced	Moderate - small *N*; controls not ADHD-screened; meds/comorbids uncontrolled; internal-only validation; ~20% attrition; single-session

Park C et al., 2023 [12]	ML detection of aggression episodes from waist-worn actigraphy in children with/without ADHD	Diagnosis / prediction - aggression/ agitation	*N*=39; ages 7–16; repeated 1-week wear (3 times/12 months)	Observational monitoring; parent episode logs as labels; Random Forest model; internal validation	ActiGraph GT3X+ (ActiGraph Corp.) — triaxial accelerometer (waist)	AUC 0.893; accuracy 0.820; recall 0.850; precision 0.802; F1 0.824. Vector-magnitude acceleration higher during aggression vs non-aggression (means 1580.7±1831.1 vs 873.3±1137.2; approx. Cohen's d ≈ 0.46 estimated, epoch-level; p=0.027)	Moderate - small *N*; parent-reported labels; epoch-level split (leakage risk)
Chen IC et al., 2024 [54]	Multimodal ADHD detection in preschoolers using wearable EEG + behavioral measures	Diagnosis - brain wave pattern during tasks	Preschoolers; ADHD/controls: 43/35 (*N*=78)	Case–control ML/DL classification (Decision Tree/Random Forest/bi-LSTM); 5-fold internal validation; ensemble model	Wearable wireless EEG (Mindo BR8; 8-ch)	Ensemble accuracy 0.974<br>Sensitivity 92.3%, specificity 90.0%<br>Effect sizes: K-CPT-2 HRT SD (ADHD 52.05 ± 8.45 vs TD 47.94 ± 6.49) Cohen's d≈0.54; HRT ISI change (52.44 ± 8.89 vs 47.69 ± 6.82) Cohen's d≈0.59	Moderate - case–control; modest *n*; ambiguous CV/hold-out; no external validation; validated scales + standard wearable EEG pipeline
Ouyang CS et al., 2020 [13]	Objective evaluation of methylphenidate effects via smartwatch accelerometry	Monitoring medication adherence	*N*=10 children with ADHD (9M/1F); mean age ≈7.4 ± 1.3 y	Pre–post within-subject (baseline vs 1-month methylphenidate 10 mg/day, weekdays); paired t-tests (Bonferroni	Garmin Vivosma rt<br>3-axis accelerometer, HRV	Variance decreased after treatment: Y-axis 4.42 ± 2.17 → 2.32 ± 0.65 (p=0.0119); Z-axis 4.09 ± 1.57 → 2.41 ± 0.81 (p=0.0140). SNAP hyperactivity reduction correlated	High -single-arm pre–post, *N* = 10, short follow-up, missing SNAP post-scores, unequal

				α=0.0167); correlation with SNAP-IV (teacher)		with Y-axis variance reduction (**r=0.605**); other subscales weak/non-significant	baseline/post recording
Huang IW et al., 2024 [57]	Assess EEG complexity (parietal fuzzy entropy) to aid ADHD diagnosis	Biomarker - brainwave pattern	Children 4–7 y; ADHD/controls: 30/30	8-ch dry-EEG headband	8-channel wireless wearable EEG	Best feature set (right occipital beta PSD + parietal FuEn) achieved accuracy = 0.90;	Moderate - *N*=60 preschoolers; wearable EEG + K-CPT; internal validation only; no follow-up
Lin JW et al., 2024 [58]	Characterize EEG functional-connectivity patterns—focusing on temporal alpha dissimilarity/coherence for potential diagnosis marker	Biomarker - brainwave pattern	*N*=72; Ages 8–16 y; ADHD/controls: 53/19	Case–control, task-evoked EEG study (visual CPT and auditory CATA tasks)	EEG sensors 16-ch EEG cap	Temporal-lobe FC in alpha during CATA was higher in TD vs. ADHD ($p < 0.05$).	Moderate - small *N*, cross-sectional; group imbalance; validated CPT/CATA + EEG coherence
Santarrosa-López I et al., 2024 [60]	Develop and validate DETEC-ADHD, a web-based application that integrates	Diagnosis - attention (brainwave pattern, + cognitive tests)	*N* = 19 (Children *n* = 10; Adults n = 9; mixed ADHD and non-ADHD)	Proof-of-concept case study; Logistic Regression model	Webapp + Muse S headband (InteraXon) — EEG (dry electrodes)	Logistic Regression: accuracy = 90%; AUC = 0.92; case-study detection rates –	Moderate - secondary datasets; scales+EEG (incorporation bias);

	machine learning with personal, clinical, psychological and EEG data to detect ADHD and its subtypes in real time.					children: 100%; adults: 90%.	internal-only validation; unclear *N*; no follow-up

Table S2. Studies about mixed-function devices (monitoring+intervention)

Study	Objective	Symptom/Target	Population	Study design	Device/Sensors involved	Key findings	Methodological concern
Ayearst LE et al., 2023 [53]	Wearable digital intervention to improve on-task behavior—specifically attention, hyperactivity/impulsivity, executive	Attention, hyperactivity/impulsivity, executive function	ADHD, 8–12 y; *N*=38 (parent raters *N*=38; teacher raters *N*=26); 4-week school wear; unmedicated	Single-arm, open-label pre–post pilot (4-week wearable use) in unmedicated children with ADHD; baseline→post	Revibe Connect (Revibe Technologies) — haptic prompts; tap-back self-reports; step logging, 3-axis accelerometer, gyroscope	Parent ADHD-RS-5 inattention d=1.07; hyperactivity/impulsivity d=0.70. Teacher ADHD-RS-5 inattention d=0.54. WFIRS-P school learning r≈0.58 (large). APRS academic	High - single-arm, unblinded; small *N*; short follow-up; industry-funded

	function, and academic performance			parent/teacher ratings; no randomization, blinding, or control		productivity d=0.59 (moderate)	
Sonne T et al., 2015 [55]	Design and preliminary evaluation of CASTT—a real-time assistive wearable to help children with ADHD regain attention in school	Attention, impulsivity, self-regulation	Children 2nd-5th grade (*n*=20, ADHD/controls: 11/9)	Non-randomized, uncontrolled observational feasibility pilot study	CASTT (Child Activity Sensing and Training Tool) custom wearable + smartphone system: Heart rate monitor, accelerometers (limbs), EEG	Improved attention tracking; usability issues	High - uncontrolled; tiny *N* (incl. single-case assistive); short sessions; manual recognition; unvalidated outcomes
Dibia V, 2016 [61]	Smartwatch app (FOQUS) to support focus and reduce anxiety in adults with ADHD/attention difficulties	Anxiety, attention	Survey *n*=27 (ages 16–40) + 7-day usability study *n*=10 (ages 21–30)	User-centred design; cognitive walkthrough + 7-day prototype usability test (no control)	Samsung Gear 2 (Samsung Electronics). PPG heart rate (pre/post meditation feedback); vibrotactile cues; positive-message priming	80% reported reduced stress/anxiety after meditation; observed HR decreases pre→post	High - uncontrolled pilot; *n*=10; 7-day window; ADHD not confirmed; self-report & consumer HR; no comparator
Leikauf JE et al., 2021 [17]	Feasibility study of an Apple Watch app, tracking movement and delivering visual/haptic	Attention, hyperactivity	ADHD; *N*=32; ages 8–17; 6-week follow-up	Open-label single-arm pilot; weekly ADHD-RS via parent report; linear mixed models for symptom	Apple Watch Series 0 (Apple Inc.) 3-axis accelerometer (actigraphy for movement);	ADHD-RS total β −1.2 units/week (95% CI −1.88 to −0.56; F=13.4; p=.0004); Inattentive β −0.8/week (p=7×10^{-5}); Hyperactive/Impulsive β −0.4/week (p=.02);	High - uncontrolled; short follow-up

	feedback to manage hyperactivity/attention in youth with ADHD			trajectories; exit interviews (feasibility/acceptability)	haptic motor (biofeedback)	no adverse events; older age associated with greater improvement	
Garcia JJ et al., 2013 [64]	Design-driven personal informatics (KITA/WRISTWIT) to support self-awareness and on-task behavior in ADHD	Hyperactivity, attention/on-task behavior, self-regulation/behavioral inhibition	Children (KITA: 4–7 yrs *N*=2, WRISTWIT: 8–12 yrs *N*=5) Context informants *N*=15	Empirical Research Through Design; iterative prototyping; in-situ school testing; exploratory sensing—no control group	KITA: waist-worn toy + "nest" (3-axis accelerometer; vibration motor; 31 LEDs; IR link; microcontroller/speaker in nest). WRISTWIT: bracelet (3-axis accelerometer; 12-LED time display)	KITA pilot: ~16% reduction in in-class activity vs baseline; high engagement reported. WRISTWIT concept: accelerometry distinguished on-/off-task states; supports time awareness.	Hgh - very small *N*; ultra-short tests; uncontrolled pre–post; prototype/Wizard-of-Oz; non-standardized outcomes; device signal issues
Whitehead JC et al., 2022 [56]	Remote EEG-neurofeedback efficacy for ADHD-related symptoms, cognition, and EEG markers	Attention/executive control, anxiety, depression, brainwave biomarker	*N*=593 (560 included), age>13<br><br>Questionnaire pre–post *n*=301; CPT pre–post *n*=99 with known ADHD status (plus *n*=104 unknown status); resting EEG baseline	Retrospective single-group pretest–posttest; home/clinic use	Muse EEG headband (InteraXon) via Myndlift app — 4 dry electrodes	(Cohen's d): questionnaires—Large pre–post improvements: ADHD-RS-IV abnormal d=2.41, GAD-7 abnormal d=1.24, PHQ-9 abnormal d=1.13, ASRS abnormal d=1.05, GHQ-12 abnormal d=0.99; CPT—response-time variability d=1.02–1.24, average RT d=0.56	High - single-arm retrospective; self-selected; short FU; in-app self-report; no control

			n=271; pre–post EEG n=41			(healthy), commission d=0.55–0.62, omission d=0.34–0.48; EEG—baseline DAR higher in abnormal ASRS (d=0.37); pre-post DAR reduced in abnormal group (d=0.70)	
Santamaría-Vázquez E. et al. 2025 [32]	Test whether combined respiratory biofeedback, neurofeedback and median nerve stimulation improve ADHD symptoms	Hyperactivity/impulsivity, anxiety/arousal, behavioral, learning, psychosomatic problems	*N*=60; ADHD randomized active group(AG)/sham group(SG): 31/29; ages 8–18;	Exploratory randomized, double-blind, sham-controlled, two-arm parallel trial; 10 sessions over 2 weeks; pre/post/1-mo follow-up; resting-state EEG	Qey-DTx NMS (median nerve stimulation) stimulator (wrist electrodes); ProComp Infiniti with respiration belt (breathing sensor); EEG Neuroamp II	Within-group improvements in AG post-treatment and at 1-mo follow-up (Cohen's d: post—hyperactivity index −0.45, anxiety −0.34, impulsivity-hyperactivity −0.40; follow-up—learning −0.62, hyperactivity index −0.50, impulsivity-hyperactivity −0.53)	Moderate - sham-controlled RCT; underpowered; short FU; parent-rated outcomes; meds/sex imbalance

Table S3. Studies about intervention-only devices

Study	Objective	Symptom/Target	Sample	Study design	Device/Sensors involved	Key findings	Methodological concern
McGough JJ et al., 2019 [66]	Non-invasive neuromodulation during sleep for symptom	Overall ADHD symptom severity (ADHD-RS)	Children 8–12 y; randomized: active/sham = 32/30 (*N*=62)	Double-blind RCT; 4 weeks nightly eTNS + 1-week blinded discontinuation;	Monarch eTNS System (NeuroSigma): external stimulator with	ADHD-RS: significant group×time ($F(1,228)=8.12$, $p=.005$); Cohen's d=0.50 at week 4.	Moderate - small *N*; short FU; industry ties;

	improvement in ADHD			weekly ADHD-RS & CGI; mechanistic qEEG	adhesive forehead patch electrodes	Clinical Global Impression-Improvement responders at week 4: 52% AG vs 14% SG (NNT=3). qEEG: increased frontal spectral power with active eTNS; partial r (EEG change ↔ ADHD-RS change) = −0.34 to −0.41	blinding/sham strong
Richter Y et al., 2023 [72]	Peripheral visual stimulation “Neuro-glasses” for adult ADHD—efficacy and safety over 2 months	Attention, executive function	ADHD, 18–40 y; enrolled *N*=108; per-protocol *N*=97; wear ≥2 h/day	Open-label single-arm clinical trial; pre–post assessments (ASRS, BRIEF-A, CPT-3); CGI-I at endpoint	Neuro-glasses (Sparkles™, VIZO Specs Ltd.) - standard lenses with semi-transparent peripheral stimuli; personalization with eye-tracking	ASRS-Inattention improved (p=0.037), Cohen’s d=0.22; BRIEF-A Metacognition improved (p=0.029), d=0.23; CPT-3 detectability d′ improved (p=0.027), d=0.23; CPT-3 commission errors reduced (p=0.004), d=0.30; 62% CGI-I responders	High - open-label, no control; CPT hardware change; sponsor-designed/analyzed; short FU
Bartlett G et al., 2024 [65]	Evaluate whether a wrist-worn haptic device (Doppel) reduces anxiety and improves focus in adults	Anxiety, attention	Adults 18–25 y with self-reported ADHD; *N*=49 at baseline; 4-week *n*=37; 8-week *n*=32	Double-blind randomized controlled trial; active HR-matched vibrations vs fixed-pattern	Doppel wristband + smartphone app; haptic actuator delivering heartbeat-like vibrations	No superiority of active vs comparator at 4 or 8 wk (all p≥.31; partial $\eta^2 \leq .03$). Time effects across groups: anxiety ↓ (η^2=.10) and focus ↑ (η^2=.22)	Moderate - small *N*; ~35% attrition; self-report outcomes; self-reported usage; Strengths:

	with ADHD over 8 weeks		(active 14 / comparator 18)	comparator; intention-to-treat			double-blind RCT with active comparator
McDermott AF et al., 2016 [70]	EEG feed-forward modeling (Atentiv/CogoLand) attention-training for pediatric ADHD; Neurofeedback training via EEG)	Attention, impulsivity, academic productivity	ADHD, 8–12 y; randomized: 46 (immediate FFM = 21; wait-list control = 19; total randomized = 46; 32M/14F)	Randomized parallel-group trial (8-week FFM vs non-pharmacological community care), waitlist crossover; outcomes at post and 3-month follow-up	EEG headband with three frontal electrodes (Zeo Sleep Manager™) + PC game (CogoLand®)	Clinician ADHD-RS: −36% vs control; partial η^2 (Group×Time)=0.434. Parent ADHD-RS: −31%; partial η^2=0.141. CGI: partial η^2 (Group×Time)=0.282. PERMP problems attempted: +26% (η^2>0.150). Effects largely maintained at 3 months; Quotient® ADHD no improvement	High - unblinded; heterogeneous control; per-protocol; small N/attrition; follow-up only after crossover
Arpaia P et al., 2020 [67]	Wearable single-channel SSVEP BCI with AR glasses for robot-based rehabilitation in ADHD; evaluate accuracy/latency and feasibility	Attention, hyperactivity, impulsivity	Algorithm tuning: *N*=20 healthy adults; Robot-control test: *N*=10 healthy adults; Clinical preliminary: *N*=4 children with ADHD (6–8 y)	Instrumentation study + observational case study; training-less single-channel SSVEP with eye-blink detection; lab evaluation and rehab-center pilot	Epson Moverio BT-200 AR glasses (eye-blink detection); Olimex EEG-SMT (single-channel EEG); Sanbot Elf robot.	Accuracy–latency trade-off (e.g., 92.6% at ~3.71 s vs 70.8% at ~0.64 s); clinical target setting selected ~1.5 s response time; case study average accuracy >83% with ITR up to 39 bits/min; preliminary ADHD tests reported positive acceptability/attentional engagement.	High - uncontrolled; small *N*; short FU; outcomes NR (validity)
Arpaia P et al., 2021 [68]	Wearable AR-based single-	Attention, hyperactivity,	Children 5–10 y; *N*=18 (ADHD);	Pilot case study (task-based	Epson Moverio BT-200 AR	Adherence: 18/18 accepted wearing;	High - uncontrolled;

	channel EEG (SSVEP) BCI to control a social robot for ADHD therapy; preliminary adherence evaluation	impulsivity, executive function, cognitive skills	plus adult benchmark *N*=10	robot control); descriptive outcomes on acceptance/adherence; no inferential testing.	glasses (eye-blink detection); Olimex EEG-SMT (single-channel EEG); Sanbot Elf robot.	completion: all 8–10 y finished tasks; some 5–7 y had ergonomics/attention issues; prior adult test accuracy ≈83.5% for command detection	*N*=18; single session (~10 min); mixed diagnosis; non-validated outcomes
Arpaia P et al., 2022 [69]	Evaluate a wearable EEG-based brain computer interface for rehabilitation/training ADHD therapy, assessing adherence and preliminary cognitive/attentional gains	Attention, hyperactivity, impulsivity, executive function, cognitive skills	Adherence/acceptability: *N*=18 ADHD children; Therapy cohort: *N*=7 ADHD children	Single-arm pilot (within-subject pre–post); task-based sessions (planning, path-following, inhibition) while controlling a social robot	Epson Moverio BT-200 AR glasses (eye-blink detection); Olimex EEG-SMT (single-channel EEG); Sanbot Elf robot.	High acceptability/adherence (18 screened). All 7 treated children showed improvement across BIA subtests after 1 month (e.g., higher semantic/phonological fluency, better visual-sequential and Span-4; fewer reading errors)	High - *n*=4 ADHD; no control; single session; non-standard clinical outcomes
Clinical trial NCT06189703 [71]	Examine the safety and effectiveness of tRNS on unmedicated pediatric patients	ADHD overall symptoms	Recruiting children (7–12 yrs)	Randomized, sham-controlled, double-blind clinical trial	Novostim 2 - Transcranial random noise stimulation device	Subjects will undergo either tRNS or sham treatment for 10 days during a two-week period in a home-simulated environment. Each treatment session is 20 minutes, during which their attention will be maintained using a software game.	

Methodological concern is a qualitative, study-level judgment (Low/Moderate/High) summarizing selection/confounding, outcome measurement validity, missing data/attrition, adherence/feasibility, and sample size/follow-up. No formal risk-of-bias tool was applied; judgments are narrative and descriptive.

- **Low**: No material threats to inference. Sampling and comparators (where relevant) are reasonable; outcomes are validated; missing data are limited/handled; adherence is documented; sample size and follow-up are adequate for the study aims.
- **Moderate**: Notable but non-fatal limitations (e.g., small or convenience sample, short follow-up, partial reporting, observational/quasi-experimental design with incomplete confounding control, consumer-grade or indirect outcomes with limited validation, or modest missing data).
- **High**: Limitations that substantially compromise inference (e.g., uncontrolled pre–post only, major confounding, non-validated outcomes for the claimed endpoint, high or differential attrition, single-session/very brief follow-up for sustained claims, unclear/biased sampling, or analysis flaws such as data leakage).

Assignment rule: If any domain is **High**, the overall rating is **High**. If none are High but ≥1 domains are **Moderate**, the rating is **Moderate**. Only if all domains are **Low** is the rating **Low**.

Abbreviations: ABCD - Adolescent Brain Cognitive Development cohort; acc - Accuracy; ADHD — Attention-Deficit/Hyperactivity Disorder; ADHD-H/I — ADHD Hyperactive/Impulsive subtype; ADHD-RS-5 — ADHD Rating Scale, 5th Edition; ADHD-RS-IV — ADHD Rating Scale, DSM-IV version; AG — Active Group; APRS — Academic Performance Rating Scale; AR — Augmented Reality; ASRS — Adult ADHD Self-Report Scale; AUC — Area Under the ROC Curve; BCI — Brain–Computer Interface; BRIEF-A — Behavior Rating Inventory of Executive Function – Adult version; CASTT — Child Activity Sensing and Training Tool; CATA — Continuous Auditory Test of Attention; CGI — Clinical Global Impression; CGI-I — Clinical Global Impression – Improvement; CI — Confidence Interval; CPT — Continuous Performance Test; CPT-3 — Conners' Continuous Performance Test, 3rd Edition; CV — Cross-Validation; DAR — (EEG) Delta/Alpha power ratio; d — Cohen's d (standardized mean difference); d′ — d-prime (signal-detection sensitivity index); DL — Deep Learning; DTx — Digital Therapeutic; EDA — Electrodermal Activity; EEG — Electroencephalography; eTNS — External Trigeminal Nerve Stimulation; F1 — F1-score (harmonic mean of precision & recall); FC — (EEG) Functional Connectivity; FFM — Feed-Forward Modeling; FPR — False Positive Rate; FU - follow-up; GAD-7 — Generalized Anxiety Disorder 7-item scale; GHQ-12 — General Health Questionnaire-12; GPS — Global Positioning System; GSR — Galvanic Skin Response; HRT — (CPT) Hit Reaction Time; HR — Heart Rate; HRV — Heart-Rate Variability; IMU — Inertial Measurement

Unit; ISI — Inter-Stimulus Interval; ITR — Information Transfer Rate (BCI metric); i-KNN — iterative/instance-based k-Nearest Neighbors (filtering step used in modeling); K-CPT-2 — Conners' Kiddie Continuous Performance Test, 2nd Edition; KNN — k-Nearest Neighbors; LGBM — Light Gradient Boosting Machine; LR — Logistic Regression; ML — Machine Learning; MNS / NMS — Median Nerve Stimulation (noted both ways in the tables); NNT — Number Needed to Treat; NPV — Negative Predictive Value; NF — Neurofeedback; PERMP — Permanent Product Measure of Performance; PHQ-9 — Patient Health Questionnaire-9; PPG — Photoplethysmography; PSD — Power Spectral Density; qEEG — Quantitative EEG; QoL — Quality of Life; RCT — Randomized Controlled Trial; RF — Random Forest; RNN — Recurrent Neural Network; RT — Reaction Time; SD — Standard Deviation; SG — Sham Group; SNAP-IV — Swanson, Nolan and Pelham Rating Scale – IV; SSVEP — Steady-State Visual Evoked Potential; SVM — Support Vector Machine; TD — Typically Developing (controls); TPR — True Positive Rate; VADPRS — Vanderbilt ADHD Diagnostic Parent Rating Scale; WFIRS-P — Weiss Functional Impairment Rating Scale – Parent report; XGB — Extreme Gradient Boosting (XGBoost); β — Beta (regression slope/standardized coefficient); η^2 (partial) — Partial eta-squared (effect-size measure); IR — Infrared (e.g., IR link in device description); LED — Light-Emitting Diode
